# Beyond Trolleyology: The CNI Model of Moral-Dilemma Responses

**DOI:** 10.1177/10888683241234114

**Published:** 2024-03-13

**Authors:** Bertram Gawronski, Nyx L. Ng

**Affiliations:** 1 The University of Texas at Austin, USA

**Keywords:** deontology, moral judgment, multinomial modeling, trolley problem, utilitarianism

## Abstract

A large body of research has investigated responses to artificial scenarios (e.g., trolley problem) where maximizing beneficial outcomes for the greater good (utilitarianism) conflicts with adherence to moral norms (deontology). The CNI model is a computational model that quantifies sensitivity to consequences for the greater good (*C*), sensitivity to moral norms (*N*), and general preference for inaction versus action (*I*) in responses to plausible moral dilemmas based on real-world events. Expanding on a description of the CNI model, the current article provides (a) a comprehensive review of empirical findings obtained with the CNI model, (b) an analysis of their theoretical implications, (c) a discussion of criticisms of the CNI model, and (d) an overview of alternative approaches to disentangle multiple factors underlying moral-dilemma responses and the relation of these approaches to the CNI model. The article concludes with a discussion of open questions and new directions for future research.

**Public Abstract:**

How do people make judgments about actions that violate moral norms yet maximize the greater good (e.g., sacrificing the well-being of a small number of people for the well-being of a larger number of people)? Research on this question has been criticized for relying on highly artificial scenarios and for conflating multiple distinct factors underlying responses in moral dilemmas. The current article reviews research that used a computational modeling approach to disentangle the roles of multiple distinct factors in responses to plausible moral dilemmas based on real-world events. By disentangling sensitivity to consequences, sensitivity to moral norms, and general preference for inaction versus action in responses to realistic dilemmas, the reviewed work provides a more nuanced understanding of how people make judgments about the right course of action in moral dilemmas.

Imagine the following scenario: A doctor contracts a lethal and highly contagious virus while working for a humanitarian aid organization in a foreign country. The doctor’s chances of surviving are very low unless the doctor is returned to their home country for special treatment. However, bringing the doctor back involves a considerable risk of causing an outbreak in their home country, potentially causing the death of many more. What is the morally right thing to do in this scenario? Returning the doctor to their home country to save their life or not returning the doctor to avoid spreading the lethal virus? Although the scenario may sound hypothetical, it describes the real case of Dr. Kent Brantly, an American doctor who got infected with Ebola in the summer of 2014 while he was working in Liberia. In the weeks before Dr. Brantly was returned to the United States and cured of the deadly disease, his case sparked heated debates in his home country about what would be the morally right decision (Blinder & Grady, 2014). While some claimed a moral duty to save Dr. Brantly’s life by returning him to the United States for treatment, others argued that not returning him is the morally right thing to do because it prevents the potential death of a larger number of people that might be caused by an Ebola outbreak in the United States.

For centuries, moral philosophers have tried to identify universal principles that could offer normative guidance in moral dilemmas like the one described in the opening paragraph. More recently, moral psychologists have taken an empirical approach to understanding people’s preferences for different options in moral dilemmas. Expanding on the latter question, the current article reviews research that has used a computational modeling approach to disentangle distinct factors underlying responses in moral dilemmas. The central idea underlying this work is that responses in moral dilemmas like the one described in the opening paragraph are jointly shaped by a person’s sensitivity to consequences for the greater good (*C*), sensitivity to moral norms of harm and care (*N*), and general preference for inaction versus action (*I*). Based on the acronyms of the three factors, the computational model used to quantify them is called the CNI model of moral-dilemma responses (Gawronski et al., 2017). Expanding on a brief overview of the work that inspired the development of the CNI model, the current article reviews (a) the core assumptions of the model, (b) empirical findings obtained with the model, (c) theoretical implications of the reviewed work, (d) criticisms raised against the model, and (e) its relation to alternative approaches. The article concludes with a discussion of open questions and new directions for future research using the CNI model.

## Positionality and Citations

To provide a context for the current review, we acknowledge relevant aspects of our identities that may have influenced this work. The first author was socialized in a Western European cultural context. He received his academic training in Germany and the United States and has held academic positions in Canada and the United States. The second author was socialized in a Southeast Asian cultural context. She received her academic training in Singapore, the United Kingdom, and the United States. Despite their different cultural and ethnic backgrounds, the two authors share the belief that the human mind is characterized by universal principles that generalize across contexts, individuals, and cultures and that universal psychological principles can produce systematic differences in behavioral phenomena across contexts, individuals, and cultures. These assumptions are also central for their work using the CNI model of moral-dilemma responses, which proposes that three general factors shape responses in moral dilemmas, although the relative impact of the three factors can vary systematically across contexts, individuals, and cultures.

The works cited in the current review include articles authored by scholars from 50 countries spreading across six continents, including Africa (i.e., Kenya), Asia (i.e., China, India, Indonesia, Iran, Japan, Kazakhstan, Lebanon, Malaysia, Pakistan, Philippines, Singapore, Thailand, Turkey, United Arab Emirates), Europe (i.e., Austria, Belgium, Bulgaria, Croatia, Czechia, Denmark, France, Germany, Greece, Hungary, Italy, Netherlands, North Macedonia, Norway, Poland, Portugal, Romania, Russia, Serbia, Slovakia, Spain, Sweden, Switzerland, Turkey, United Kingdom), North America (i.e., Canada, Mexico, United States), Oceania (i.e., Australia, New Zealand), and South America (i.e., Argentina, Brazil, Chile, Colombia, Ecuador, Peru).

## Trolleyology and Its Limitations

A paradigmatic feature of research on moral-dilemma responses is the use of hypothetical scenarios that pit moral norms against consequences for the greater good. The most well-known example is the trolley problem, in which a runaway trolley is said to approach a group of five people who would be killed if the trolley continues its path. In a variant called the *switch dilemma*, participants are told that pulling a lever would redirect the trolley to a different track where it would kill only one person instead of five (Foot, 1967). In a variant called the footbridge dilemma, participants are told that pushing a man from a bridge would kill the man but save the five people by stopping the trolley (Thomson, 1976). Adopting terminology from moral philosophy, judgments supporting either of the two actions (i.e., pulling the lever, pushing the man) have been described as characteristically utilitarian in the sense that they maximize wellbeing for the larger number of people (i.e., killing one saves the lives of five; see Conway et al., 2018); judgments opposing the two actions have been described as characteristically deontological in the sense that such judgments conform to a relevant moral norm (i.e., the norm that one should not kill others; see Conway et al., 2018). Although researchers have used a variety of structurally similar dilemmas to study moral judgments (see Christensen et al., 2014), the trolley problem is by far the most frequently used scenario, which inspired the term *trolleyology* as an umbrella label for research that has used the trolley problem and structurally similar scenarios to investigate moral-dilemma responses (Greene, 2023).

Despite its popularity, trolleyology research has been criticized for multiple reasons, a common concern being the implausible and unrealistic nature of the employed scenarios (e.g., Bauman et al., 2014; Körner et al., 2019). Although this concern could be addressed by using more plausible dilemmas based on historical real-world events (e.g., Körner & Deutsch, 2023), there is a broader methodological concern that still applies to research using more plausible, realistic dilemmas, which is that the trolleyology paradigm includes two confounds that undermine unambiguous interpretations of the obtained results.

The first confound is rooted in the treatment of utilitarian and deontological judgments as opposite ends of a bipolar continuum, in that endorsement of the outcome-maximizing option necessarily involves a rejection of the norm-adhering option, and vice versa (see Conway & Gawronski, 2013). To illustrate the significance of this confound, consider the finding that people with high levels of psychopathy tend to show a stronger preference for utilitarian over deontological judgments in the trolley problem than people with low levels of psychopathy (e.g., Bartels & Pizarro, 2011; Glenn et al., 2010; Kahane et al., 2015; Paruzel-Czachura & Farny, 2024; Patil, 2015; for a meta-analysis, see Marshall et al., 2018). Does this mean that people high in psychopathy care more about maximizing wellbeing than people low in psychopathy? Based on what is known about psychopathy (see Hare & Neumann, 2008; Patrick, 2022), this seems rather unlikely. Instead, it seems more likely that people high in psychopathy care less about causing harm, which increases their willingness to accept harmful actions in the trolley problem. This example illustrates that a stronger preference for utilitarian over deontological judgments in the trolleyology paradigm may reflect either (a) a stronger concern about outcomes or (b) a weaker concern about norm violations (or both). Because endorsement of the utilitarian option is confounded with a rejection of the deontological option (and vice versa), it is not possible to distinguish between the two cases.

The second confound is that the utilitarian option typically involves action (e.g., pulling the switch, pushing the man), and the deontological option typically involves inaction (e.g., not pulling the switch, not pushing the man), which conflates moral codes with general action tendencies (see Crone & Laham, 2017). To illustrate the significance of this confound, consider a modified version of the footbridge dilemma in which a man is about to fall off the bridge in front of the trolley, which would kill the man and save the five people by stopping the trolley. Yet, the man could be saved by grabbing his arm. In this dilemma, the mapping of moral codes with action and inaction is reversed (as in the Ebola scenario described in the opening paragraph), in that wellbeing for the larger number of people would be maximized by inaction (i.e., not grabbing the man’s arm) while adherence to moral norms would suggest action (i.e., grabbing the man’s arm). Yet, consistency of utilitarian and deontological responses across the two dilemma variants is not a necessary given. For example, while some participants may support the norm-adhering deontological option in both the original footbridge dilemma (i.e., not pushing the man) and its modified variant (i.e., grabbing the man’s arm), others may support inaction in both cases (i.e., not pushing the man; not grabbing the man’s arm). The latter response pattern could be described as an instance of the omission bias (see Cushman et al., 2006; Spranca et al., 1991), which refers to the finding that harm caused via action is often perceived as worse than the same harm caused via inaction (e.g., killing someone vs. letting someone die; for a meta-analysis, see Yeung et al., 2022). This asymmetry in the perception of harm can lead people to show a general preference for inaction irrespective of the particular situation. Yet, the psychological processes underlying a general preference for inaction (e.g., asymmetric perceptions of harm caused by action versus inaction) are presumably different from those underlying unconditional support of norm-adhering options. In the trolleyology paradigm, it is not possible to distinguish between the two cases because moral codes are confounded with general action tendencies.

In sum, the two confounds in the trolleyology paradigm render empirical findings ambiguous because differences in moral-dilemma judgments may reflect (a) differences in the tendency to make outcome-maximizing judgments, (b) differences in the tendency to make norm-conforming judgments, or (c) differences in general action tendencies (or any combination of the three).

## The CNI Model

The CNI model is a computational model that resolves these ambiguities by separately quantifying sensitivity to consequences for the greater good (*C*), sensitivity to moral norms of harm and care (*N*), and general preference for inaction versus action (*I*) in responses to moral dilemmas (Gawronski et al., 2017). To this end, the model uses responses across four types of moral dilemmas that differ in terms of two factors: (a) whether the benefits of a focal action for the greater good are either greater or smaller than its costs and (b) whether the focal action is either prohibited by a proscriptive norm or prescribed by a prescriptive norm. The three factors are quantified by estimating (a) the extent to which participants’ responses are influenced by the manipulation of cost-benefit ratios (i.e., sensitivity to consequences), (b) the extent to which participants’ responses are influenced by the manipulation of norm type (i.e., sensitivity to moral norms), and (c) the extent to which participants prefer inaction (vs. action) responses irrespective of cost-benefit ratios and norm type (i.e., general preference for inaction versus action). A sample dilemma in its four variants—inspired by the Ebola dilemma described in the opening paragraph—is presented in Table 1. The initial battery of CNI dilemmas created by Gawronski et al. (2017) included six basic dilemmas in four variants (comprising a total of 24 dilemmas); an extended battery of 12 basic dilemmas in four variants (comprising a total of 48 dilemmas) was presented by Körner et al. (2020). Addressing concerns about low plausibility and low realism in trolleyology research (e.g., Bauman et al., 2014; Körner et al., 2019), all CNI dilemmas are based on real-world events that have been the subject of viral debates (see Gawronski et al., 2017; Körner et al., 2020). Examples of events that served as inspiration for the CNI dilemmas include Dr. Kent Brantly’s Ebola’s infection in Liberia and the debate about his return to the United States for treatment (immune-deficiency dilemma); the physician-assisted suicide of brain-cancer patient Brittany Maynard (assisted-suicide dilemma); the beheading of American journalist James Foley by the terrorist group ISIS (abduction dilemma); recurring debates about a potential denial of life support to obtain organs for transplants (transplant dilemma); the use of torture to gain information about the location of an abducted child in Canada (torture dilemma); and debates about the potential use of unapproved vaccines to fight the Ebola epidemic in various African countries (vaccine dilemma).

**Table 1.** Example of a Moral Dilemma Involving Either a Proscriptive or a Prescriptive Norm Where the Benefits of Action Are Either Greater or Smaller Than the Costs of Action.

Proscriptive norm prohibits action		Prescriptive norm prescribes action	
Benefits of action greater than costs	Benefits of action smaller than costs	Benefits of action greater than costs	Benefits of action smaller than costs
You are the director of a hospital in a developing country. A foreign student who is volunteering in the country got infected with a rare virus. The virus is highly contagious and deadly to seniors and children. The only medication that can effectively stop the virus from spreading has severe side-effects. Although the virus will not kill her, the student suffers from a chronic immune deficiency that will make her die from these side-effects. Would you give the student the medication in this case?	You are the director of a hospital in a developing country. A foreign student who is volunteering in the country got infected with a rare virus. The virus is highly contagious and can cause severe stomach cramps. The only medication that can effectively stop the virus from spreading has severe side-effects. Although the virus will not kill her, the student suffers from a chronic immune deficiency that will make her die from these side-effects. Would you give the student the medication in this case?	You are the director of a hospital in a developing country. A foreign student who is volunteering in the country got infected with a rare virus. The virus is highly contagious and can cause severe stomach cramps. The student suffers from a chronic immune deficiency that will make her die from the virus if she is not returned to her home country for special treatment. However, taking her out of quarantine involves a considerable risk that the virus will spread. Would you take the student out of quarantine to return her to her home country for treatment in this case?	You are the director of a hospital in a developing country. A foreign student who is volunteering in the country got infected with a rare virus. The virus is highly contagious and deadly to seniors and children. The student suffers from a chronic immune deficiency that will make her die from the virus if she is not returned to her home country for special treatment. However, taking her out of quarantine involves a considerable risk that the virus will spread. Would you take the student out of quarantine to return her to her home country for treatment in this case?

*Source.* Dilemmas adapted from Gawronski et al. (2017). Reprinted with permission from the American Psychological Association.

**Figure 1.**
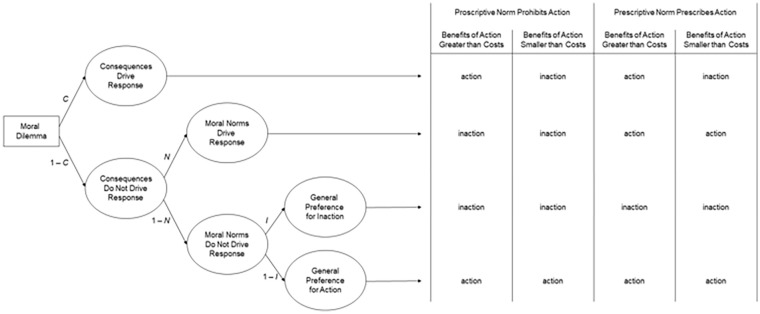
Processing Tree of the CNI Model of Moral-Dilemma Responses Predicting Action Versus Inaction Responses in Moral Dilemmas With Proscriptive and Prescriptive Norms and Consequences Involving Benefits of Action That Are Either Greater or Smaller Than the Costs of Action. *Source.* Adapted from Gawronski et al. (2017). Reprinted with permission from the American Psychological Association.

To quantify the three factors underlying responses to moral dilemmas, the CNI model uses multinomial processing tree (MPT) modeling (see Batchelder & Riefer, 1999; Erdfelder et al., 2009; Hütter & Klauer, 2016). The processing tree of the CNI model is depicted in Figure 1. Each row on the right side of the figure depicts a distinct pattern of responses across the four types of dilemmas. The three factors are captured by corresponding parameters that reflect the probability of a specific pattern in participants’ responses across the four types of dilemmas. The CNI model’s *C* parameter quantifies sensitivity to consequences as the probability of supporting action when the benefits of action are greater than the costs and supporting inaction when the benefits of action are smaller than the costs (first row in Figure 1). The CNI model’s *N* parameter quantifies sensitivity to moral norms as the probability of supporting inaction when a proscriptive norm prohibits action and supporting action when a prescriptive norm prescribes action (second row in Figure 1). The CNI model’s *I* parameter quantifies general preference for inaction versus action as the probability of supporting inaction (versus action) across all four dilemma variants (third and fourth rows in Figure 1). Each parameter can vary between 0 and 1. Higher scores on the *C* and *N* parameters reflect greater sensitivity to consequences and greater sensitivity to moral norms, respectively. For the *I* parameter, scores higher than 0.5 reflect a general preference for inaction; scores lower than 0.5 reflect a general preference for action.

Numerical scores for the three parameters are estimated via four non-redundant mathematical equations derived from the processing tree in Figure 1. These equations include the three model parameters as unknowns and the observed probabilities of support for action (versus inaction) in each of the four dilemma types as known numerical values (see Appendix).^1^ Numerical scores for the three parameters are generated via maximum-likelihood statistics, such that the discrepancy between the empirically observed probabilities of action (versus inaction) in the four dilemma variants and the probabilities predicted via the model equations is minimized. The adequacy of the model in describing the empirically observed response patterns can be evaluated via goodness-of-fit statistics, in that poor model fit indicates a large discrepancy between the response probabilities predicted by the model and the observed response probabilities in the data. Differences between groups of participants on a given parameter (e.g., differences between two experimental conditions) can be tested by constraining the parameter to be equal across groups and comparing the fit of the constrained model to the fit of the original unconstrained model. To the extent that constraining a given parameter to be equal across groups leads to a significant reduction in model fit, it can be inferred that the parameter is significantly different across groups (see Gawronski et al., 2017). To the extent that the number of observed responses for each participant is sufficiently large, individual differences along the three factors can be gauged by fitting the model to the data of each participant. The resulting scores can then be used to analyze relations to other variables (see Körner et al., 2020).^2^

Aside from a few exceptions, the CNI model has shown adequate goodness-of-fit across studies (see Gawronski et al., 2020). To date, the model has been successfully used with university students (e.g., Zhang et al., 2018), military cadets (e.g., Cornwell & Bella, 2022), participants recruited from the local community (e.g., Paruzel-Czachura et al., 2023), and workers on various crowdsourcing platforms such as Amazon’s MTurk (e.g., Gawronski et al., 2017), Prolific Academic (e.g., Ng et al., 2022), CloudResearch (e.g., Luke & Gawronski, 2021a), Lancers (Speckmann et al., 2024), SurveySwap (e.g., Oldham, 2022), and Questant (e.g., Qian et al., 2023). CNI model studies have been conducted with samples from four continents (i.e., Asia, Europe, North America, Oceania) and various countries including Australia (e.g., Halliwell, 2021), China (e.g., S. Li et al., 2020), Germany (e.g., Kroneisen & Heck, 2020), Iran (e.g., Barabadi et al., 2023), Japan (e.g., Qian et al., 2023), Poland (e.g., Białek et al., 2019), the United Kingdom (e.g., Ng et al., 2023), and the United States (e.g., Gawronski et al., 2017). Evidence regarding the reliability of the three CNI model parameters was provided in a longitudinal study with American MTurk workers (Luke & Gawronski, 2022b), showing high test–retest correlations over 1 month for the *C* parameter (*r* = .81) and *N* parameter (*r* = .84). Temporal stability was lower for the *I* parameter (*r* = .41), partly because the parameter showed lower internal consistencies at both time points (Cronbach’s *α*s = .53 and .37, respectively) than the *C* parameter (Cronbach’s *α*s = .69 and .73, respectively) and *N* parameter (Cronbach’s *α*s = .78 and .74, respectively).

**Figure 2.**
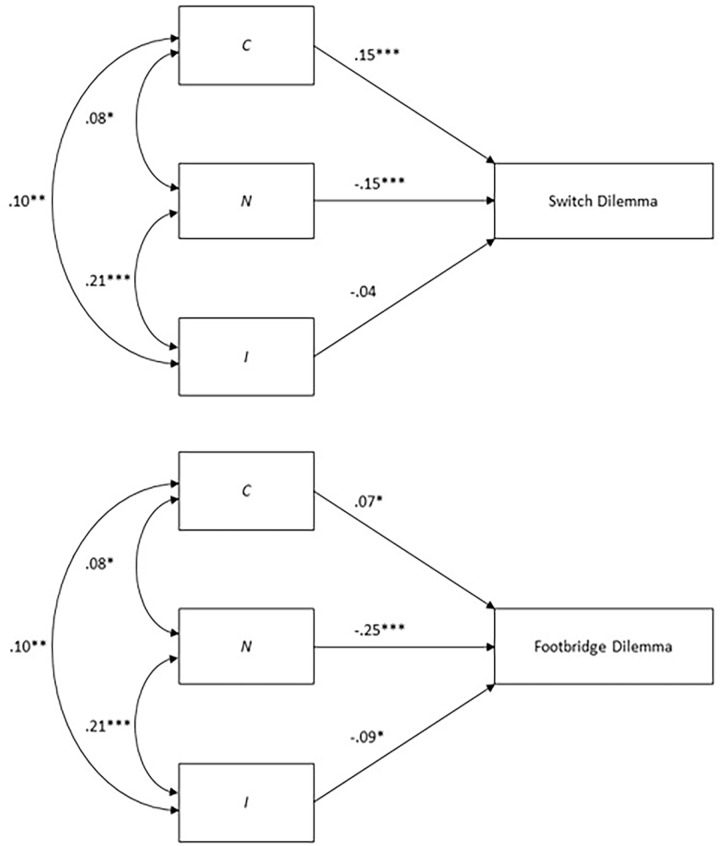
Results of Multiple-Regression Analyses Predicting Preference for Utilitarian Over Deontological Judgments in the Switch and Footbridge Variants of the Trolley Problem Via Sensitivity to Consequences (C), Sensitivity to Moral Norms (N), and General Preference for Inaction Versus Action (I). *Note.* The figure depicts the results of secondary analyses of data (*N* = 702) from Paruzel-Czachura and Farny (2024).

Although the CNI dilemmas are based on real-world events (see Gawronski et al., 2017; Körner et al., 2020), the three factors captured by the CNI model also provide valuable insights into the determinants of responses in the highly artificial trolley problem. Conceptually, stronger sensitivity to consequences on the *C* parameter should be associated with a stronger preference for utilitarian over deontological judgments; stronger sensitivity to moral norms on the *N* parameter should be associated with a weaker preference for utilitarian over deontological judgments; and stronger general preference for inaction versus action on the *I* parameter should be associated with a weaker preference for utilitarian over deontological judgments (see Gawronski et al., 2020). A secondary analysis of data from Paruzel-Czachura and Farny (2024) largely support these predictions, but the analysis also revealed some interesting differences between different variants of the trolley problem (see Figure 2).^3^ In the study by Paruzel-Czachura and Farny, Polish online participants responded to a battery of dilemmas for research using the CNI model (Körner et al., 2020), as well as the switch and footbridge versions of the trolley problem. Multiple-regression analyses using the three CNI model parameters as predictors and action-ratings on the two variants of the trolley problem as outcomes confirmed the predicted associations for the *C* and *N* parameters. While the *C* parameter showed a significantly stronger association with preference for utilitarian over deontological judgments in the switch dilemma than in the footbridge dilemma, the *N* parameter showed the opposite pattern.^4^ Moreover, the *I* parameter showed a significant negative association with preference for utilitarian over deontological judgments only for the footbridge dilemma, but not for the switch dilemma.^5^ Although the lack of a significant association in the latter case might be due to the lower reliability of the *I* parameter (see Luke & Gawronski, 2022b), these findings suggest that sensitivity to consequences might play a stronger role for responses in the switch dilemma, whereas sensitivity to moral norms and general preference for inaction versus action might play a stronger role for responses in the footbridge dilemma. These differences might explain why preference for utilitarian over deontological judgments is typically weaker in the footbridge dilemma than in the switch dilemma (see Greene, 2008) and why some studies found effects only on one type of dilemma but not the other (e.g., Suter & Hertwig, 2011; Valdesolo & DeSteno, 2006).^6^

To avoid potential misinterpretations of the constructs captured by the three CNI model parameters, we underscore three important conceptual aspects of the CNI model. First, the three parameters capture patterns of responses across different types of dilemmas at the behavioral level; they do not capture latent processes at the mental level (see Gawronski & Bodenhausen, 2015). The CNI model does not include any claims about the mental processes underlying the responses patterns captured by the three parameters (Gawronski et al., 2018). For example, the *C* parameter captures the extent to which participants support action when the benefits of action are greater than the costs and support inaction when the benefits of action are smaller than the costs. However, the model does not make any theoretical assumptions about the mental processes underlying the response pattern captured by the *C* parameter (e.g., controlled cognitive analyses of costs and benefits). Similarly, the *N* parameter captures the extent to which participants support inaction when a proscriptive norm prohibits action and support action when a prescriptive norm prescribes action. However, the model does not make any theoretical assumptions about the mental processes underlying the response pattern captured by the *N* parameter (e.g., conscious consideration of moral norms). Research using the CNI model can provide valuable evidence for testing hypotheses about the mental processes underlying moral-dilemma judgments, but the model itself does not include any assumptions about underlying mental processes. We return to this question when we discuss theoretical implications of the reviewed work.

Second, it is worth noting that the term moral norm could be interpreted in a very broad manner that would subsume the response patterns of all three parameters, not just the *N* parameter. For example, the response pattern captured by the *C* parameter could be said to reflect the moral norm “always maximize benefits” (Hennig & Hütter, 2020), and the response pattern captured by the *I* parameter could be said to reflect the moral norm “first, do no harm,” which can lead to a general aversion toward action (Baron & Goodwin, 2020). These potential interpretations raise the question of the kinds of proscriptive and prescriptive norms involved in the response pattern captured by the *N* parameter. To answer this question, it is critical to consider how the two kinds of norms are operationalized in the moral dilemmas created for research using the CNI model, and how this operationalization differs from the operationalizations underlying the other two parameters (for a detailed discussion, see Gawronski et al., 2020). For each dilemma scenario in the CNI model moral-dilemma battery (e.g., immune-deficiency scenario), there are four dilemma variants. Each of the four dilemma variants includes a pair of options involving an action and an inaction that have the same proximal effect (e.g., killing Person A and letting Person A die both result in the loss of Person A’s life). By virtue of their proximal effect, the identified action is conceptually linked to a proscriptive norm (i.e., killing someone is morally prohibited), while the opposite of the identified inaction is conceptually linked to a prescriptive norm (i.e., saving someone’s life is morally prescribed). Consequences are operationalized via the combination of proximal and distal effects, with distal effects subsuming all secondary outcomes beyond the proximal effect (e.g., sacrificing Person A’s life may save the lives of multiple others). The critical aspect in this operationalization of consequences is whether the focal action involves aggregate benefits for overall wellbeing that are either greater or smaller than aggregate costs (i.e., how many lives are saved and how many lives are lost overall?). Thus, while the *N* parameter captures the extent to which responses are shaped by the described proximal effects (i.e., support for inaction when action causes proximal harm and support for action when action prevents proximal harm; see second row in Figure 1), the *C* parameter captures the extent to which moral-dilemma responses are sensitive to overall outcomes including both proximal and distal effects (i.e., support for action when the benefits of action are greater than the costs, and support for inaction when the benefits of action are smaller than the costs; see first row in Figure 1). These two response patterns are distinct from the one captured by the *I* parameter, which reflects the extent to which people show a general preference for inaction (vs. action) regardless of the proximal and distal effects of action (see third and fourth rows in Figure 1). It is important to keep these conceptual meanings of the three parameters in mind when interpreting empirical results obtained with the CNI model.

Third, it is worth noting that, while the outcome-sensitive response pattern captured by the *C* parameter fits uniquely to the central ideas of utilitarianism, the response patterns captured by the *N* and the *I* parameters could both be described as deontological (see Baron & Goodwin, 2020; Gawronski et al., 2017, 2020). While the response pattern captured by the *N* parameter can be described as deontological for its conformity to the described operationalization of proscriptive and prescriptive norms, the response pattern captured by the *I* parameter can be described as deontological for its conformity to the general norm “first, do no harm,” which is associated with a general aversion toward action (Baron & Goodwin, 2020). However, lumping the two parameters under the undifferentiated umbrella term deontological responding seems problematic because they capture distinct patterns of responses that (a) can be influenced differently by the same factor and (b) presumably have distinct psychological underpinnings. Thus, although we continue to use the expression “preference for utilitarian over deontological judgments” to describe responses in moral dilemmas that have the same structure as the trolley problem, we avoid references to deontology in discussions of findings with the CNI model.

## Empirical Findings

Because the trolleyology paradigm confounds multiple distinct factors in the measurement of moral-dilemma responses (see Conway & Gawronski, 2013; Crone & Laham, 2017), findings obtained with this paradigm are generally ambiguous. The CNI model addresses this concern by disentangling sensitivity to consequences, sensitivity to moral norms, and general preference for inaction versus action in responses to moral dilemmas. By relying on plausible dilemmas based on real-world events (see Gawronski et al., 2017; Körner et al., 2020), research using the CNI model also addresses concerns about low plausibility and low realism in trolleyology research (e.g., Bauman et al., 2014; Körner et al., 2019).

In this section, we review empirical findings obtained with the CNI model and the insights gained from these findings. To ensure comprehensiveness of our review, we conducted a Google Scholar search (May 13, 2023) to identify all articles that cited either the original CNI model article of Gawronski et al. (2017) (*N* = 196) or the extended battery of moral dilemmas for research using the CNI model by Körner et al. (2020) (*N* = 56).^7^ We then screened the resulting list for articles that used the CNI model in at least one empirical study and excluded all articles that merely cited the two papers without reporting CNI model data. We also excluded one article that investigated presumed moral-dilemma decisions by other people rather than personal choices (Gawronski, 2022) and three articles that used modified versions of the CNI model instead of the original model (Hennig & Hütter, 2020, 2021; Skovgaard-Olsen & Klauer, 2023).^8^ Together with the original CNI model article of Gawronski et al. (2017), two manuscripts from our lab that were under editorial review at the time of our search (Ng, Luke, Gawronski, 2024; Ng, Neumann, et al., 2024), and two manuscripts in preparation (Ng, Nahon, & Gawronski, 2024; Speckmann et al., 2024), the final list included 33 papers reporting 67 independent studies with a total of 16,532 participants. A comprehensive list of these studies is provided in Table 2. The table also includes information about sample sizes, sample characteristics, and measured or manipulated variables.

**Table 2.** Comprehensive List of CNI Model Studies Reviewed in the Current Article.

Authors	Year	Study #	*N*	Sample country	Sample type	Variables
Barabadi et al.	2023	1	461	Iran	University Students	Foreign Language, Religiosity
Bialek et al.	2019	1	634	Poland	University Students	Foreign Language
Brannon et al.	2019	1	200	USA	University Students	Exogenous Testosterone, Endogenous Testosterone
Cornwell & Bella	2022	1	190	USA	Military Cadets	Regulatory Mode
Cornwell & Bella	2022	2	200	USA	Mturk Workers	Regulatory Mode
Gawronski et al.	2017	1a	201	USA	Mturk Workers	Gender
Gawronski et al.	2017	1b	197	USA	Mturk Workers	Gender
Gawronski et al.	2017	2a	194	USA	Mturk Workers	Cognitive Load
Gawronski et al.	2017	2b	194	USA	Mturk Workers	Cognitive Load
Gawronski et al.	2017	3a	186	USA	Mturk Workers	Question Frame
Gawronski et al.	2017	3b	189	USA	Mturk Workers	Question Frame
Gawronski et al.	2017	4a	184	USA	Mturk Workers	Psychopathy
Gawronski et al.	2017	4b	198	USA	Mturk Workers	Psychopathy
Gawronski et al.	2018	1a	128	USA	University Students	Happiness
Gawronski et al.	2018	1b	120	USA	University Students	Happiness
Gawronski et al.	2018	2a	119	USA	University Students	Sadness
Gawronski et al.	2018	2b	120	USA	University Students	Sadness
Gawronski et al.	2018	3a	120	USA	University Students	Anger
Gawronski et al.	2018	3b	120	USA	University Students	Anger
Gawronski & Brannon	2020	1a	140	USA	University Students	Social Power
Gawronski & Brannon	2020	1b	120	USA	University Students	Social Power
Gawronski & Brannon	2020	2a	91	USA	University Students	Social Power
Gawronski & Brannon	2020	2b	120	USA	University Students	Social Power
Gawronski & Brannon	2020	3	255	USA	University Students	Social Power
Halliwell	2021	1	130	Australia	University Students	Perceived Intent, Anger
Körner et al.	2020	1a	161	USA	Mturk Workers	Psychopathy, Empathic Concern, Need for Cognition, Instrumental Harm, Impartial Beneficence, Behavioral Activation/Inhibition, Moral Identity, Religiosity
Körner et al.	2020	1b	177	USA	Mturk Workers	Psychopathy, Empathic Concern, Need for Cognition, Instrumental Harm, Impartial Beneficence, Behavioral Activation/Inhibition, Moral Identity, Religiosity
Körner et al.	2020	2a	196	USA	Mturk Workers	Psychopathy, Empathic Concern, Need for Cognition, Instrumental Harm, Impartial Beneficence, Behavioral Activation/Inhibition, Moral Identity, Religiosity
Körner et al.	2020	2b	189	USA	Mturk Workers	Psychopathy, Empathic Concern, Need for Cognition, Instrumental Harm, Impartial Beneficence, Behavioral Activation/Inhibition, Moral Identity, Religiosity
Körner et al.	2020	S1	185	USA	Mturk Workers	Question Frame
Körner et al.	2020	S2	182	USA	Mturk Workers	Question Frame
Kroneisen & Heck	2020	1	142	Germany	Internet Communities	HEXACO Traits
Kroneisen & Heck	2020	2	249	Germany	Internet Communities	HEXACO Traits
Kroneisen & Steghaus	2021	1	199	Germany	Local Community	Processing Time
Kroneisen & Steghaus	2021	2	168	Germany	Local Community	Processing Instructions
S. Li et al.	2020	1	362	China	University Students	Psychopathy
Z. Li et al.	2021	1	78	China	University Students	Acute Stress
Lu et al.	2022	1	139	China	Local Community	Internet Addiction
Luke & Gawronski	2021a	2	242	United Kingdom	Prolific Workers	Political Orientation
Luke & Gawronski	2021a	3	506	USA	CloudResearch Workers	Political Orientation
Luke & Gawronski	2021b	1	337	USA	Mturk Workers	Psychopathy
Luke & Gawronski	2021a	1	248	USA	Mturk Workers	Political Orientation
Luke et al.	2022	1	443	USA	Prolific Workers	Psychopathy
Luke & Gawronski	2022b	1	195	USA	Mturk Workers	Temporal Stability, Big Five Personality Traits
Luke & Gawronski	2022a	1	250	USA	University Students	Big Five Personality Traits
Luke & Gawronski	2022a	2	240	USA	Mturk Workers	Big Five Personality Traits
Nadarevic et al.	2021	1	64	Germany	University Students	Foreign Language
Nadarevic et al.	2021	2	505	Germany	Internet Communities	Foreign Language
Ng et al.	2022	1	273	USA	Prolific Workers	Dishonest Behavior, Psychopathy, HEXACO Humility
Ng et al.	2022	2	270	USA	Prolific Workers	Dishonest Behavior, Psychopathy, HEXACO Humility
Ng et al.	2023	1	173	United Kingdom	Prolific Workers	Uncertainty
Ng et al.	2023	2	169	United Kingdom	Prolific Workers	Uncertainty
Ng et al.	2023	3	170	United Kingdom	Prolific Workers	Uncertainty
Ng et al.	2023	4	588	United Kingdom	Prolific Workers	Uncertainty, Question Frame
Ng, Luke, & Gawronski	2024	1	165	United Kingdom	Prolific Workers	Reasons
Ng, Luke, & Gawronski	2024	2	249	United Kingdom	Prolific Workers	Reasons
Ng, Luke, & Gawronski	2024	3	503	United Kingdom	Prolific Workers	Reasons, Processing Instructions
Ng, Nahon, & Gawronski	2024	1	479	United Kingdom	Prolific Workers	Psychopathy
Ng, Neumann, et al.	2024	1	676	United Kingdom	Prolific Workers	Psychopathy, Dark & Light Traits
Oldham	2022	1	184	Germany, United Kingdom	SurveySwap	Foreign Language
Paruzel-Czachura et al.	2023	1	329	Poland	Local Community	Alcohol, Instrumental Harm, Impartial Beneficence, Cognitive Reflection Test
Paruzel-Czachura & Farny	2024	1	702	Poland	Internet Communities	Psychopathy, Instrumental Harm, Impartial Beneficence
Quian et al.	2023	1	411	China, Japan	University Students, Questant Workers	Culture, Gender
Speckmann et al.	2024	1	392	USA, Japan	Mturk Workers, Lancers Workers	Culture
Yang et al.	2022	1	170	China	University Students	Guilt
Yang & Guo	2023	1	164	China	University Students	Envy
Zhang et al.	2018	1	197	China	University Students	Chronic Stress

In cases where we found two or more papers on a given effect that collectively report at least four independent effect sizes, we calculated meta-analytic effect sizes across studies using a random-effects model to gauge the reliability and average size of the identified effects. In such cases, we entered the relevant effect sizes reported in a given paper to a common database. If no effect sizes were reported, we calculated the relevant effect sizes from other data reported in the paper. If a paper did not include any data required to calculate effect sizes, we contacted the corresponding author with a request for the required data. In the latter two cases, all effect sizes were calculated based on means, standard errors, and sample sizes using the online companion to the *Practical Guide to Meta-Analyses* by Lipsey and Wilson (2001) at https://www.campbellcollaboration.org/escalc/html/EffectSizeCalculator-SMD8.php. To obtain a common metric for experimental and correlational studies, effects sizes included as Cohen’s *d*s in our database were converted to Pearson correlations using the R package *effectsize* version 0.8.3 (Ben-Shachar et al., 2020). The meta-analyses were conducted using the R package *meta* version 6.2-1 (Balduzzi et al., 2019). Interpretations of effect sizes are based on the benchmarks recommended by J. Cohen (1988), with *r* = .10 as the benchmark for a small effect, *r* = .30 as the benchmark for a medium effect, and *r* = .50 as the benchmark for a large effect. All data and codes for the reported meta-analyses are available at the Open Science Framework (OSF) at https://osf.io/2pdnz/.

### Judgments vs. Decisions

An important distinction in the moral-dilemma literature pertains to the difference between moral judgments and moral decisions (Pletti et al., 2017; Tassy et al., 2013). In studies on moral judgments, participants are asked to evaluate the described actions (e.g., is it acceptable to do X?); in studies on moral decisions, participants are asked to indicate whether they would perform the described action (e.g., would you do X?). Although most trolleyology studies focus on either one or the other, a small number of studies that directly compared judgments and decisions has found inconsistent effects of question type at the overall sample level (e.g., Pletti et al., 2017; Tassy et al., 2013).

**Figure 3.**
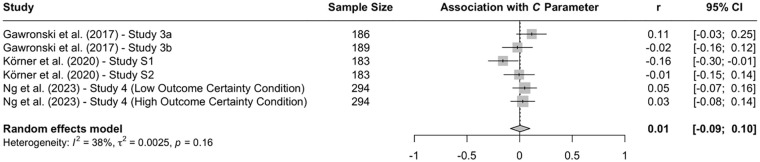
Effects of Response Type (Moral Judgment vs. Moral Decision) on Sensitivity to Consequences. *Note.* Effect sizes are depicted as Pearson correlations. Higher correlations reflect higher parameter scores in the moral-decision condition than in the moral-judgment condition. Error bars depict 95% confidence intervals.

**Figure 4.**
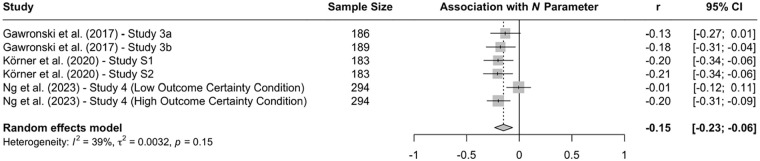
Effects of Response Type (Moral Judgment vs. Moral Decision) Sensitivity to Moral Norms. *Note.* Effect sizes are depicted as Pearson correlations. Higher correlations reflect higher parameter scores in the moral-decision condition than in the moral-judgment condition. Error bars depict 95% confidence intervals.

**Figure 5.**
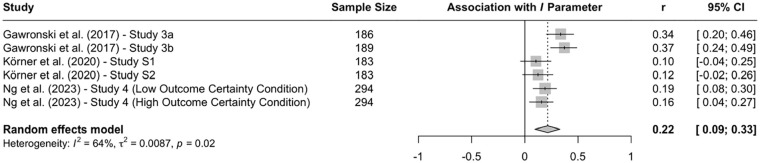
Effects of Response Type (Moral Judgment vs. Moral Decision) on General Preference for Inaction Versus Action. *Note.* Effect sizes are depicted as Pearson correlations. Higher correlations reflect higher parameter scores in the moral-decision condition than in the moral-judgment condition. Error bars depict 95% confidence intervals.

Research using the CNI model revealed more consistent evidence for systematic differences across question types. In a direct comparison of moral judgments and moral decisions, two studies with American MTurk workers found that participants showed stronger sensitivity to moral norms in moral judgments than to moral decisions (Gawronski et al., 2017, Experiments 3a and 3b). Conversely, general preference for inaction versus action was stronger in moral decisions than moral judgments. These differences replicated in two studies with American MTurk workers (Körner et al., 2020) and one study with Prolific workers from the United Kingdom (Ng et al., 2023). A meta-analysis on all available CNI model studies that directly compared moral judgments and moral decisions confirmed the obtained differences on the *N* and *I* parameters (see Figures 3–5), indicating that sensitivity to moral norms is significantly weaker for moral decisions than moral judgments (*r* = −.150, 95% CI [−.234, −.064], *t* = −4.45, *p* = .007) while general preference for inaction versus action is significantly stronger for moral decisions than for moral judgments (*r* = .215, 95% CI [.094, .330], *t* = 4.52, *p* = .006). Sensitivity to consequences does not significantly differ between moral judgments and moral decisions in the meta-analytic comparison (*r* = .005, 95% CI [−.087, .098], *t* = 0.15, *p* = .889). Together, these findings suggest that people tend to be more action-averse and less sensitive to proscriptive and prescriptive norms about proximal harm when they make decisions about how to respond as opposed to when they make abstract judgments about the moral acceptability of actions. Because the two effects of question type compensate each other when outcome-maximizing actions are pit against norm-adhering inactions, effects of question type can be difficult to detect with the trolleyology paradigm, which may falsely suggest that there are no systematic differences between moral judgments and moral decisions. Yet, research using the CNI model clearly shows that moral-dilemma responses differ depending on whether participants are asked to make a moral judgment or a moral decision.

### Cognitive Reflection

A common assumption in the trolleyology literature is that utilitarian judgments are the product of controlled cognitive analyses of costs and benefits (Greene, 2008). This assumption is consistent with findings of trolleyology studies suggesting that (a) time pressure reduces preference for utilitarian over deontological judgments (e.g., Suter & Hertwig, 2011), (b) cognitive load increases response times for utilitarian judgments (e.g., Greene et al., 2008), and (c) individuals with a stronger (vs. weaker) propensity to engage in cognitive reflection show stronger preference for utilitarian over deontological judgments (e.g., Patil et al., 2021). However, a closer examination of the relevant literature suggests that the available evidence is much less consistent than suggested by the selective citation patterns in this area, in that the findings of multiple studies do not align with the idea that utilitarian judgments are the product of controlled cognitive analyses of costs and benefits (e.g., Bago & De Neys, 2019; Gürçay & Baron, 2017; Hashimoto et al., 2022; Körner & Volk, 2014; Stanley et al., 2018; Tinghög et al., 2016; Trémolière & Bonnefon, 2014).

Research using the CNI model reveals a similarly mixed picture. Consistent with the assumption that utilitarian judgments are the product of controlled cognitive analyses of costs and benefits (Greene, 2008), a study with German participants from the local community found that sensitivity to consequences was attenuated under time pressure (Kroneisen & Steghaus, 2021). Correspondingly, a study with Polish participants from the local community found that sensitivity to consequences was stronger among participants who showed high (vs. low) scores on the Cognitive Reflection Test (Paruzel-Czachura et al., 2023). However, counter to these findings, two studies with American MTurk workers found that cognitive load increased general preference for inaction versus action without affecting sensitivity to consequences and sensitivity to moral norms (Gawronski et al., 2017, Experiments 2a and 2b). Moreover, sensitivity to moral norms has been found to be stronger among American MTurk workers with a high need for cognition (Körner et al., 2020) and among British Prolific workers who were asked to think about reasons for their choices (Ng, Luke, & Gawronski, 2024). Mere instructions to think carefully about the dilemmas (versus respond quickly and intuitively) showed no significant effects on any of the three CNI model parameters in a sample of German participants from the local community (Kroneisen & Steghaus, 2021).

**Figure 6.**
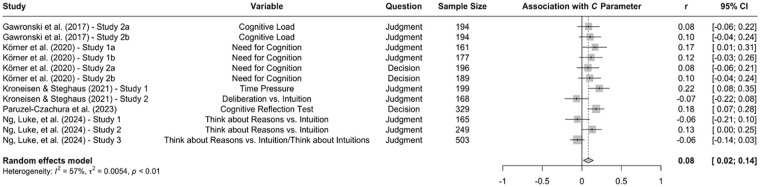
Effects of Cognitive Reflection on Sensitivity to Consequences. *Note.* Effect sizes are depicted as Pearson correlations. Higher correlations reflect a positive association between cognitive reflection and a given parameter score. Error bars depict 95% confidence intervals.

**Figure 7.**
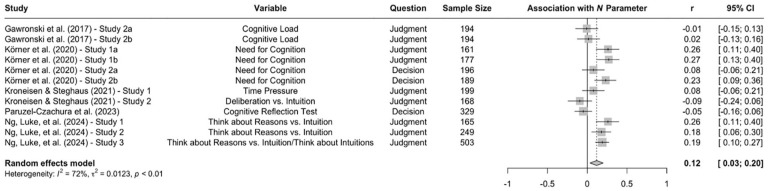
Effects of Cognitive Reflection on Sensitivity to Moral Norms. *Note.* Effect sizes are depicted as Pearson correlations. Higher correlations reflect a positive association between cognitive reflection and a given parameter score. Error bars depict 95% confidence intervals.

**Figure 8.**
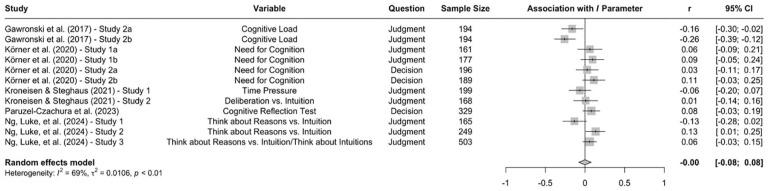
Effects of Cognitive Reflection on General Preference for Inaction Versus Action. *Note.* Effect sizes are depicted as Pearson correlations. Higher correlations reflect a positive association between cognitive reflection and a given parameter score. Error bars depict 95% confidence intervals.

A meta-analysis on the data from these studies revealed a significant effect of cognitive reflection on the *C* and *N* parameters, but no significant effect on the *I* parameter (see Figures 6–8). Consistent with the assumption that utilitarian judgments are the product of controlled cognitive analyses of costs and benefits (Greene, 2008), sensitivity to consequences significantly increased as a function of cognitive reflection. However, the size of the obtained association was rather small and below the benchmark for a small effect (*r* = .082, 95% CI [.020, .143], *t* = 2.90, *p* = .014). A slightly larger effect emerged for sensitivity to moral norms, which also increased as a function of cognitive reflection (*r* = .118, 95% CI [.035, .200], *t* = 3.12, *p* = .010). Cognitive reflection showed no meta-analytic association with general preference for inaction versus action (*r* = −.001, 95% CI [−.080, .078], *t* = −0.03, *p* = .978). Together, these results suggest that cognitive reflection increases sensitivity to consequences as well as sensitivity to moral norms, although the impact of cognitive reflection on either factor is relatively small overall.

Although the conceptualization of cognitive reflection in our meta-analysis is in line with the modal view in the literature, a study by Ng, Luke, and Gawronski (2024) suggests that it may be important to distinguish between degree and content of cognitive reflection. While degree of cognitive reflection refers to the mental resources that are available or invested to process relevant information, content of cognitive reflection refers to the specific information that is being processed. According to Ng, Luke, and Gawronski (2024), degree and content are conceptually distinct because one can devote more or less resources for the processing of any type of information, irrespective of its content. For example, people can spend more or less time thinking about their gut reactions, just as they can spend more or less time thinking about reasons that would justify a particular choice. Consistent with the distinction between degree and content, three studies with British Prolific workers found that sensitivity to moral norms was stronger when participants were instructed to think about reasons for their choices than when they were instructed to respond intuitively or to think about their intuitions (Ng, Luke, & Gawronski, 2024). Importantly, thinking about reasons increased sensitivity to moral norms irrespective of processing time. There were no reliable effects of thinking about reasons on sensitivity to consequences and general preference for inaction versus action. Because degree and content are not separated in most experimental manipulations (e.g., cognitive load, time pressure) and individual-difference measures (e.g., Need for Cognition Scale, Cognitive Reflection Test) of cognitive reflection, future research on the role of cognitive reflection in moral-dilemma responses may benefit from using alternative approaches that permit analyses of their distinct roles.

### Psychopathy

A well-replicated finding in the trolleyology literature is that high levels of psychopathy are associated with a stronger preference for utilitarian over deontological judgments (e.g., Bartels & Pizarro, 2011; Glenn et al., 2010; Kahane et al., 2015; Paruzel-Czachura & Farny, 2024; Patil, 2015; for a meta-analysis, see Marshall et al., 2018). Research using the CNI model suggests a more complex relation between psychopathy and moral-dilemma responses. A first set of two studies with American MTurk workers found negative associations between psychopathy and all three CNI model parameters, indicating that participants with high (vs. low) levels of psychopathy showed (a) weaker sensitivity to consequences, (b) weaker sensitivity to moral norms, and (c) weaker general preference for inaction versus action (Gawronski et al., 2017, Experiments 4a and 4b). This finding has been replicated for both moral judgments and moral decisions with American MTurk workers (e.g., Körner et al., 2020; Luke & Gawronski, 2021b) and Prolific workers from the United Kingdom (Ng et al., 2022, Study 2). However, while the negative association between psychopathy and sensitivity to moral norms seems to be very robust, some studies did not replicate the negative association with sensitivity to consequences in samples of Chinese university students (Z. Li et al., 2021), American Prolific workers (Luke et al., 2022), British Prolific workers (Ng et al., 2022; Ng, Nahon, & Gawronski, 2024; Ng, Neumann, et al., 2024), and Polish participants recruited online (Paruzel-Czachura & Farny, 2024). Yet other studies did not replicate the negative association with general preference for inaction versus action in samples of Chinese university students (Z. Li et al., 2021), American Prolific workers (Luke et al., 2022), and British Prolific workers (Ng, Neumann, et al., 2024).

**Figure 9.**
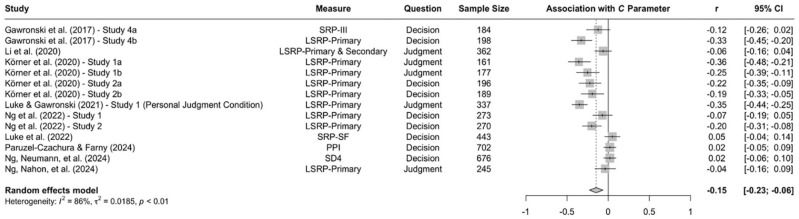
Associations of Psychopathy With Sensitivity to Consequences. *Note.* Effect sizes are depicted as Pearson correlations. Higher correlations reflect a positive association between psychopathy and a given parameter score. Error bars depict 95% confidence intervals.

**Figure 10.**
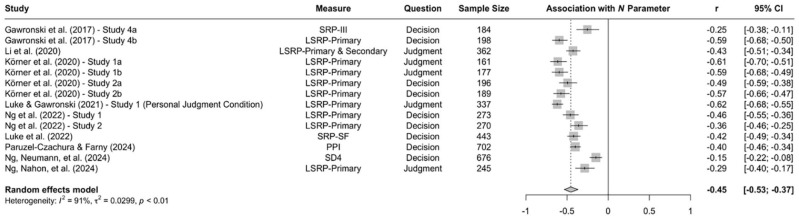
Associations of Psychopathy With Sensitivity to Moral Norms. *Note.* Effect sizes are depicted as Pearson correlations. Higher correlations reflect a positive association between psychopathy and a given parameter score. Error bars depict 95% confidence intervals.

**Figure 11.**
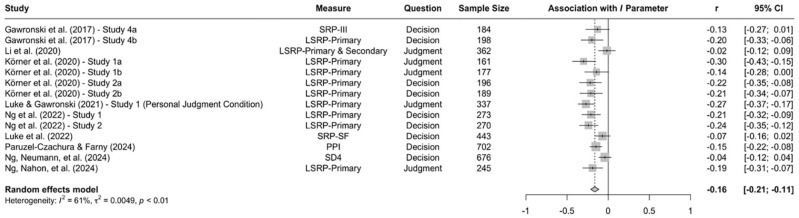
Associations of Psychopathy With General Preference for Inaction Versus Action. *Note.* Effect sizes are depicted as Pearson correlations. Higher correlations reflect a positive association between psychopathy and a given parameter score. Error bars depict 95% confidence intervals.

When the available data are submitted to a meta-analysis, psychopathy shows significant negative associations with all three parameters (see Figures 9–11).^9^ While the meta-analytic associations are relatively small for sensitivity to consequences (*r* = −.147, 95% CI [−.229, −.062], *t* = −3.74, *p* = .003) and general preference for inaction versus action (*r* = −.163, 95% CI [−.213, −.113], *t* = −6.92, *p* < .001), the meta-analytic association with sensitivity to moral norms (*r* = −.454, 95% CI [−.534, −.366], *t* = −9.98, *p* < .001) is close to the benchmark for a large effect (see J. Cohen, 1988). Together, these findings corroborate the conclusion that high levels of psychopathy are associated with (a) weaker sensitivity to consequences, (b) weaker sensitivity to moral norms, and (c) weaker general preference for inaction versus action. Among the three associations, the most notable is the negative association between psychopathy and sensitivity to consequences, which stands in contrast to a common inference from trolleyology findings that individuals high in psychopathy are more utilitarian than individuals low in psychopathy (see Marshall et al., 2018). If anything, the findings obtained with the CNI model suggest the opposite.

A pertinent question in the forensic and clinical literatures is why individuals with high levels of psychopathy show enhanced antisocial behaviors (Hare & Neumann, 2008; Leistico et al., 2008; Patrick, 2022). Some argue that individuals high in psychopathy differ from those low in psychopathy in terms of their understanding of what society considers morally right or wrong (e.g., Blair, 1995; Blair et al., 1995). Yet, others argue that individuals high and low in psychopathy have a similar understanding of societal conventions about right or wrong but instead differ in terms of their personal standards about the acceptability of certain actions (e.g., Aharoni et al., 2012, 2014; Cima et al., 2010). A study with American MTurk workers suggests that both factors play a role in moral-dilemma responses (Luke & Gawronski, 2021b). Participants with different levels of psychopathy were presented with a battery of moral dilemmas for research using the CNI model and asked either (a) whether they personally find the described action acceptable (personal-judgment condition) or (b) whether society would find the described action acceptable (societal-judgment condition). The rationale underlying this manipulation is that, if associations between psychopathy and moral-dilemma responses are driven by differences in the understanding of what society considers right or wrong, correlations between psychopathy and moral-dilemma responses should not differ between the two experimental conditions. In contrast, if individuals high and low in psychopathy have a similar understanding of societal conventions about right or wrong, but instead differ in terms of their personal standards about the acceptability of certain actions, psychopathy should be correlated with moral dilemma judgments in the personal-judgment condition, but not in the societal-judgment condition (see Luke & Gawronski, 2021b).

For sensitivity to consequences, the results suggest that differences between individuals high versus low in psychopathy are primarily driven by differences in their understanding of societal conventions. In contrast, for general preference for inaction versus action, individuals high and low in psychopathy seem to have a similar understanding of societal conventions, but instead differ in terms of their personal standards for acceptable actions. Finally, for sensitivity to moral norms, differences between individuals high versus low in psychopathy seem to be driven by both differences in the understanding of societal conventions and differences in personal standards for acceptable actions. With respect to sensitivity to moral norms, a study with American Prolific workers further suggests that differences between individuals high versus low in psychopathy are driven by the affective and interpersonal facets of psychopathy (Luke et al., 2022). The lifestyle and antisocial facets of psychopathy seem to play a less significant role. While the affective facet includes characteristics such as remorselessness and callousness, the interpersonal facet includes characteristics such as pathological lying tendencies and manipulativeness. The less-relevant lifestyle facet includes characteristics such as irresponsibility and impulsivity, and the antisocial facet includes characteristics such as delinquency and behavioral issues (Hare & Neumann, 2008).

In sum, findings obtained with the CNI model suggest that individuals high in psychopathy are not “more utilitarian” than individuals low in psychopathy, as it has been inferred from trolleyology studies. If anything, individuals high in psychopathy show a weaker sensitivity to consequences for the greater good than individuals low in psychopathy—in addition showing a weaker sensitivity to moral norms and a weaker general preference for inaction versus action. Moreover, the obtained associations between psychopathy and the three aspects of moral-dilemma responses seem to be rooted in a combination of (a) differences in the understanding of what society considers morally right or wrong and (b) differences in personal standards about the acceptability of certain actions, with the affective and interpersonal facets of psychopathy being more relevant for the obtained differences in moral-dilemma responses than the lifestyle and antisocial facets.

### Empathic Concern

Given that individuals high in psychopathy often show deficits in empathy (e.g., Ali et al., 2009), one factor that may be relevant for the obtained associations between psychopathy and moral-dilemma responses is empathic concern. Consistent with this idea, several studies found that lower levels of empathic concern are associated with a greater preference for utilitarian over deontological judgments in the trolleyology paradigm (e.g., Gleichgerrcht & Young, 2013; for a meta-analysis, see Nasello & Triffaux, 2023), mirroring the positive association between psychopathy and preference for utilitarian over deontological judgments (for a meta-analysis, see Marshall et al., 2018). Expanding on these findings, four studies with American MTurk workers (Körner et al., 2020) found that higher levels of empathic concern were reliably associated with stronger sensitivity to moral norms on the CNI model’s *N* parameter, and this association replicated for moral judgments and moral decisions. Empathic concern showed no reliable associations with sensitivity to consequences and general preference for inaction versus action. These findings suggest that prior evidence for a negative association between empathic concern and preference for utilitarian over deontological judgments in the trolleyology paradigm is driven by stronger sensitivity to moral norms among individuals with high empathic concern. In addition, the findings suggest that empathic concern may be a central factor underlying sensitivity to moral norms as operationalized in the CNI model (i.e., support for inaction when action causes proximal harm and support for action when action prevents proximal harm), which has important implications for understanding the processes underlying moral-dilemma responses.

### Moral Beliefs

In addition to the critiques reviewed earlier in this article, trolleyology research has been criticized for focusing exclusively on people’s willingness to cause sacrificial harm for the greater good. Kahane et al. (2018) argued that the dominant focus on sacrificial harm is incomplete because it ignores a prosocial component that is fundamental to utilitarianism: the impartial concern for the well-being of everyone, regardless of closeness or proximity. To address this concern, Kahane et al. (2018) developed a two-dimensional self-report measure called the Oxford Utilitarianism Scale (OUS) which is supposed to capture both components of utilitarianism. One subscale of the OUS measures self-reported permissive attitudes toward sacrificial harm, called Instrumental Harm (IH); the other subscale measures self-reported impartial concern for the greater good, called Impartial Beneficence (IB). Consistent with the concerns of Kahane et al. (2018), the researchers found a positive association between IH and preference for utilitarian over deontological judgments in the trolleyology paradigm, but moral-dilemma responses showed no reliable association with IB.

The distinction between IH and IB raises the question of how the two components of utilitarianism are related to the three factors captured by the CNI model. If the critique of Kahane et al. is correct, one might expect a positive association between IH and sensitivity to consequences, with IB showing no reliable associations with any of the three factors. Consistent with these predictions, a study with Polish participants from the local community found no significant associations between IB and any of the three CNI model parameters for moral decisions (Paruzel-Czachura et al., 2023). Moreover, a significant positive association between IH and the *C* parameter indicated that participants who reported more permissive attitudes toward instrumental harm showed stronger sensitivity to consequences in moral decisions. Yet, IH also showed a significant negative association with the *N* parameter, indicating that participants who reported more permissive attitudes toward sacrificial harm showed weaker sensitivity to moral norms.

**Figure 12.**
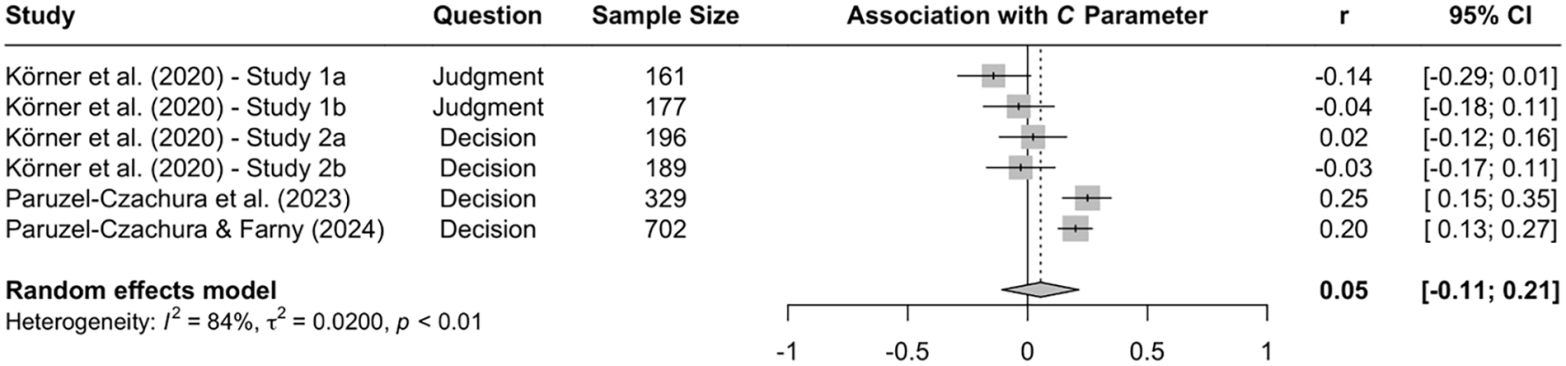
Associations of Instrumental Harm With Sensitivity to Consequences. *Note.* Effect sizes are depicted as Pearson correlations. Higher correlations reflect a positive association between instrumental harm and a given parameter score. Error bars depict 95% confidence intervals.

**Figure 13.**
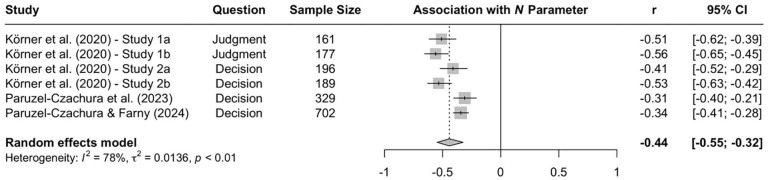
Associations of Instrumental Harm With Sensitivity to Moral Norms. *Note.* Effect sizes are depicted as Pearson correlations. Higher correlations reflect a positive association between instrumental harm and a given parameter score. Error bars depict 95% confidence intervals.

**Figure 14.**
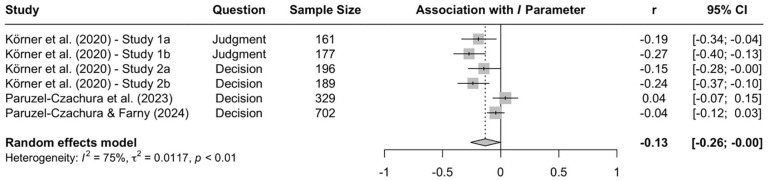
Associations of Instrumental Harm With General Preference for Inaction Versus Action. *Note.* Effect sizes are depicted as Pearson correlations. Higher correlations reflect a positive association between instrumental harm and a given parameter score. Error bars depict 95% confidence intervals.

**Figure 15.**
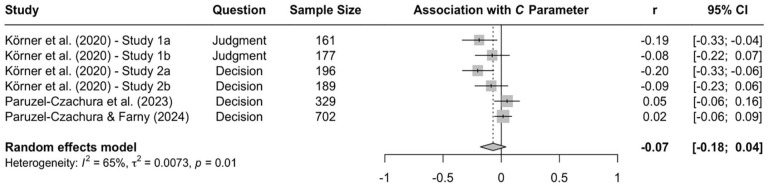
Associations of Impartial Beneficence With Sensitivity to Consequences. *Note.* Effect sizes are depicted as Pearson correlations. Higher correlations reflect a positive association between impartial beneficence and a given parameter score. Error bars depict 95% confidence intervals.

**Figure 16.**
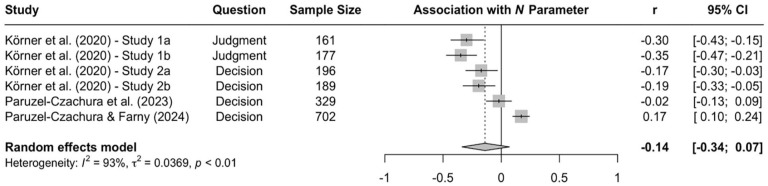
Associations of Impartial Beneficence With Sensitivity to Moral Norms. *Note.* Effect sizes are depicted as Pearson correlations. Higher correlations reflect a positive association between impartial beneficence and a given parameter score. Error bars depict 95% confidence intervals.

**Figure 17.**
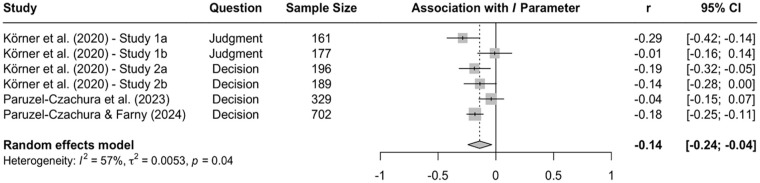
Associations of Impartial Beneficence With General Preference for Inaction Versus Action. *Note.* Effect sizes are depicted as Pearson correlations. Higher correlations reflect a positive association between impartial beneficence and a given parameter score. Error bars depict 95% confidence intervals.

When all available data are considered, the picture seems much more complex than suggested by the critique of trolleyology research by Kahane et al. (2018). At the time we prepared the current article, the available evidence included one study with Polish participants from the local community (Paruzel-Czachura et al., 2023), four studies with American MTurk workers (Körner et al., 2020), and one study with Polish online participants (Paruzel-Czachura & Farny, 2024). Among the six relations between the three CNI model parameters and the two OUS dimensions, three results stand out (see Figures 12–17). First, neither IH (*r* = .055, 95% CI [−.107, .214], *t* = 0.87, *p* = .426) nor IB (*r* = −.070, 95% CI [−.179, .040], *t* = −1.64, *p* = .164) showed a significant positive association with sensitivity to consequences. Similar to the common finding in attitude research that people often say one thing but then do something else (Ajzen, 1991; Fazio, 1990; Wicker, 1969), these results suggest that self-reported concerns about the greater good do not necessarily translate into outcome-maximizing choices. Second, the largest association found was a negative relation between IH and sensitivity to moral norms (*r* = −.442, 95% CI [−.547, −.324], *t* = −8.79, *p* < .001). Along with the non-significant associations with the *C* parameter, this result suggests that permissive attitudes toward sacrificial harm increases people’s willingness to violate moral norms; yet they seem less likely to reflect genuine utilitarian concerns about the greater good. Third, both IH (*r* = −.134, 95% CI [−.259, −.004], *t* = −2.65, *p* = .046) and IB (*r* = −.140, 95% CI [−.241, −.036], *t* = −3.45, *p* = .018) showed a significant negative association with general preference for inaction versus action. To the extent that general action aversion can be the product of concerns about potentially harmful actions (see Baron & Goodwin, 2020), these associations suggest that the two dimensions influence moral-dilemma responses via reduced aversion to potentially harmful actions. Interestingly, this seems to be the case not only for the IH dimension but also for the IB dimension of the OUS.

Together, these findings raise important questions about the validity of the critique of moral-dilemma research by Kahane et al. (2018) and the relation between moral beliefs and moral behavior. First, counter to the claim of Kahane et al., impartial concern for the well-being of everyone does matter for moral-dilemma responses, as suggested by the obtained association between IB and general preference for inaction versus action. Second, echoing insights from the literature on attitude-behavior relations, the finding that neither IH nor IB showed a reliable positive association with sensitivity to consequences suggests that self-reported moral beliefs do not necessarily translate into corresponding choices. Instead, it seems that permissive attitudes toward sacrificial harm merely increase people’s willingness to violate moral norms (as suggested by the reliable negative association between IH and sensitivity to moral norms). Neither permissive attitudes toward sacrificial harm nor self-reported impartial concern for the well-being of everyone seem to promote choices that maximize well-being in a utilitarian sense (as suggested by the lack of reliable associations of IH and IB with sensitivity to consequences).

### Moral Identity

Another type of moral belief that has been found to be associated with different factors underlying moral-dilemma responses is the extent to which being a moral person is central to one’s self-concept. This aspect of the self-concept has been called self-importance of moral identity internalization (Aquino & Reed, 2002), which we refer to as moral identity for the sake of brevity. While moral identity has been found to predict different kinds of moral behavior (for a meta-analysis, see Hertz & Krettenauer, 2016), we are not aware of any trolleyology research that investigated associations between moral identity and preference for utilitarian over deontological judgments. Yet, in four CNI model studies with American MTurk workers, moral identity has been found to be positively associated with sensitivity to consequences as well as sensitivity to moral norms, and these associations emerged for both moral judgments and moral decisions (Körner et al., 2020). An interesting aspect of this finding is that the two associations have compensatory effects on preference for utilitarian over deontological judgments, which can lead to an overall null effect if they are similar in size. While the positive association with sensitivity to consequences should lead to a positive association between moral identity and preference for utilitarian over deontological judgments, the positive association with sensitivity to moral norms should lead to a negative association between moral identity and preference for utilitarian over deontological judgments. These compensatory effects are the reason why associations between moral identity and moral-dilemma responses can remain undetected in the trolleyology paradigm. Similar to the multifaceted effects of question framing (i.e., moral judgments vs. moral decisions), the findings obtained for moral identity illustrate how the confounds in trolleyology research can conceal complex associations that can be uncovered with advanced data analytic techniques like the CNI model.

### Incidental Emotions

While integral emotions are emotional states elicited by a focal stimulus, incidental emotions are emotional states aroused by factors other than the focal stimulus, but that may nevertheless influence responses to the focal stimulus (see Cameron et al., 2013). Prior trolleyology research suggests that incidental happiness increases preference for utilitarian over deontological judgments (Valdesolo & DeSteno, 2006). The finding has been interpreted as evidence for the idea that deontological judgments are rooted in automatic emotional reactions to the idea of causing harm (see Greene et al., 2001). Specifically, happiness has been assumed to dampen negative emotional reactions, which in turn increases preference for utilitarian over deontological judgments by reducing deontological tendencies.

Research using the CNI model revealed conceptual problems with this interpretation. Two studies with American undergraduate students that used musical clips to induce either a happy or neutral emotional state found that participants who were presented with happy music while making moral judgments showed weaker sensitivity to moral norms than participants who were presented with neutral music (Gawronski et al., 2018). At first glance, this finding may seem consistent with the suggested interpretation of prior trolleyology findings, in that sensitivity to moral norms clearly reflects an instance of deontological responding. However, from the perspective of the CNI model, the concept of negative emotional reactions to the idea of causing harm is most closely related to general preference for inaction versus action, in that such negative emotional reactions should increase action aversion regardless of the specific situation. It seems less clear how negative emotional reactions to the idea of causing harm could produce a preference for inaction when a proscriptive norm prohibits action and, at the same time, a preference for action when a prescriptive norm prescribes action, as captured by the CNI model’s *N* parameter (see Gawronski et al., 2018). From this perspective, it seems more likely that incidental happiness influences moral-dilemma responses by dampening negative emotional reactions to the idea of violating moral norms (see Nichols & Mallon, 2006). Although this explanation may seem very similar to the original account in terms of automatic emotional responses to the idea of causing harm, the two are different in important ways because the original explanation explicitly dismissed moral norms as a relevant factor (see Greene et al., 2001). We will return to this point when we discuss the theoretical implications of the reviewed work.

Additional findings by Gawronski et al. (2018) suggest that the observed effect of happiness is emotion-specific rather than valence-driven, in that sadness and anger do not produce corresponding effects in the opposite direction. Using a similar manipulation to induce either a sad or neutral emotional state, two studies with American undergraduate students and an integrative analysis of the data from both studies (see Curran & Hussong, 2009) found no significant effect of sadness on any of the three CNI model parameters. The same was true for two studies with American undergraduate students that used an anger-evoking (vs. neutral) musical clip to manipulate incidental anger.

Although the reviewed evidence may suggest that moral-dilemma responses are affected only by positive emotions (e.g., happiness) but not by negative emotions (e.g., sadness, anger), a study with Chinese university students found a significant effect for the negative emotion of envy (Yang & Guo, 2023). In this study, half of the participants completed an immersive thinking-and-writing task (Polman & Ruttan, 2012) involving a competitive situation arousing envy. The remaining half completed a neutral thinking-and-writing task about aspects of the lab room. Afterwards, participants made moral judgments on a series of dilemmas for research using the CNI model. The results revealed a significant effect of envy on the *N* parameter, indicating that participants in the envy group showed a lower sensitivity to moral norms than participants in the control group. There were no significant effects on sensitivity to consequences and general preference for inaction versus action.

Further evidence for effects of negative emotions comes from a study with Chinese university students that investigated effects of guilt (Yang et al., 2022). In this study, half of the participants were asked to recall and write about a personal event that made them feel guilty. The remaining half were asked to write about aspects of the lab room. Afterwards, participants made moral judgments on a series of dilemmas for research using the CNI model. The results revealed a significant effect of guilt on the *N* parameter, indicating that participants in the guilt group showed a higher sensitivity to moral norms than participants in the control group. There were no significant effects on sensitivity to consequences and general preference for inaction versus action.

Together with the reviewed null effects of sadness and anger (Gawronski et al., 2018), the findings obtained for envy (Yang & Guo, 2023) and guilt (Yang et al., 2022) speak against a simple dimensional interpretation that attributes effects of incidental emotions to their positive versus negative valence. Although effects of incidental emotions seem to be outcome-specific in the sense that they uniquely affect sensitivity to moral norms without affecting sensitivity to consequences and general preference for inaction versus action, effects of incidental emotions seem to be emotion-specific and independent of valence, in that (a) both positive emotions (e.g., happiness) and negative emotions (e.g., guilt) can increase sensitivity to moral norms, and (b) negative emotions can either increase (e.g., guilt) or decrease (e.g., envy) sensitivity to moral norms.

### Stress

Beyond the reviewed effects of incidental emotions, some studies suggest that stress can influence moral-dilemma responses (Z. Li et al., 2021; Zhang et al., 2018). Prior trolleyology research has found that stress induced via the Trier Social Stress Test (TSST; Kirschbaum et al., 1993) reduced preference for utilitarian over deontological judgments (Youssef et al., 2012). Similar to the interpretation originally proposed for the effect of happiness (Valdesolo & DeSteno, 2006), this effect has been interpreted as being driven by enhanced emotional reactions to the idea of causing harm, which may be heightened by stress. Consistent with this interpretation and the argument that negative emotional reactions to the idea of causing harm should increase general action aversion (see Gawronski et al., 2018), a study with Chinese university students found that acute stress induced via the TSST increased general preference for inaction versus action in moral judgments (Z. Li et al., 2021). In addition, acute stress increased participants’ sensitivity to moral norms. However, a related study investigating effects of chronic stress in a sample of Chinese university students found only a positive association between chronic stress and general preference for inaction versus action in moral judgments; there was no significant association between chronic stress and sensitivity to moral norms (Zhang et al., 2018). Together, these results suggest that stress can influence moral-dilemma responses by increasing general action aversion, consistent with the ideas (a) that stronger emotional reactions to the idea of causing harm may increase action aversion in moral dilemmas and (b) stress may increase emotional reactions to the idea of causing harm.

### Social Power

Common wisdom holds that power corrupts. However, extant theories suggest that this idea oversimplifies the complex relation between power and morality, in that power can either decrease or increase moral behavior (Lammers et al., 2015; Scholl et al., 2022). As for effects of power on moral-dilemma responses, different theories of power lead to different predictions about how power may influence sensitivity to consequences, sensitivity to moral norms, and general preference for inaction versus action.

First, socio-structural theories of power suggest that being in a high-power position enhances people’s preference for rules because rules protect one’s high status in the social hierarchy (Lammers & Stapel, 2009). Conversely, being in a low-power position is assumed to enhance people’s focus on outcomes to detect potential negative effects of the current hierarchy. Together, these assumptions suggest that (a) high (vs. low) power should increase sensitivity to moral norms on the CNI model’s *N* parameter and that (b) high (vs. low) power should decrease sensitivity to consequences on the CNI model’s *C* parameter.

Second, theories of power that address effects on basic information processing suggest that feelings of high power are associated with more abstract construals of judgment-relevant information (e.g., Magee et al., 2010; P. K. Smith & Trope, 2006). Together with findings suggesting that abstract construals enhance the focus on outcomes in a utilitarian sense (e.g., Aguilar et al., 2013; Amit & Greene, 2012), these theories imply that high (vs. low) power should increase sensitivity to consequences on the CNI model’s *C* parameter, opposite to one of the predictions by socio-structural theories.

A third category of theories suggests that high power makes people resistant to social influence, in that they become less likely to attend to the feelings and perceptions of others (e.g., Galinsky et al., 2006; Magee & Smith, 2013). As a result of these mechanisms, high power can reduce empathic concern and potential concerns about norm violations, which should be reflected in weaker sensitivity to moral norms on the CNI model’s *N* parameter under conditions of high (vs. low) power, opposite to one of the predictions by socio-structural theories.

Finally, theories of power focusing on action tendencies suggest that high power increases the likelihood of engaging in focal actions by enhancing approach tendencies and reducing behavioral inhibition (e.g., Galinsky et al., 2003; Hirsh et al., 2011; Keltner et al., 2003). According to this perspective, power should influence moral-dilemma responses via general action tendencies, in that high (vs. low) power should decrease general preference for inaction versus action on the CNI model’s *I* parameter.

A series of five experiments with American undergraduate students revealed mixed evidence for these hypotheses, in that high (vs. low) power increased sensitivity to moral norms in one set of studies and decreased sensitivity to moral norms in another set of studies (Gawronski & Brannon, 2020). The critical moderator of the observed effect was whether power was manipulated via (a) randomly assigned social roles in a simulated supervisor-intern role-play (see Anderson & Berdahl, 2002) or (b) a memory task that asked participants to recall a personal experience involving high or low power (see Galinsky et al., 2003). Consistent with one of the two predictions implied by social-structural theories (Lammers & Stapel, 2009), participants assigned to a high-power role showed stronger sensitivity to moral norms in moral judgments than participants assigned to a low-power role. Moreover, consistent with theories suggesting that high power reduces attention to the feelings and perceptions of others (e.g., Galinsky et al., 2006; Magee & Smith, 2013), participants who recalled a personal experience involving high power showed weaker sensitivity to moral norms in moral judgments than participants who recalled a personal experience involving low power. The diverging effects of the two manipulations echo concerns in the broader literature on social power that understanding the multifaceted effects of power requires a more nuanced distinction between structural and psychological aspects of power (Galinsky et al., 2015; Tost, 2015). The reviewed findings with the CNI model indicate that structural aspects of power (e.g., differential power associated with social roles) can influence moral-dilemma responses in a manner that is diametrically opposite to the effects of psychological power (e.g., differential feelings of power induced via memory recall).

### Testosterone

A similar pattern of diametrically opposite effects was found in a registered report on the impact of testosterone levels on moral decisions (Brannon et al., 2019). An earlier trolleyology study by Carney and Mason (2010) found that higher levels of endogenous testosterone are associated with a stronger preference for utilitarian over deontological judgments. Expanding on these findings, Brannon et al. (2019) aimed to investigate whether this association is driven by (a) stronger sensitivity to consequences, (b) weaker sensitivity to moral norms, or (c) weaker general preference for inaction versus action resulting from high testosterone levels. Either of the three cases is consistent with prior findings in the testosterone literature. First, high levels of testosterone have been found to be associated with high reward sensitivity (e.g., Campbell et al., 2010) and a strong focus on outcomes (e.g., Coates & Herbert, 2008), which suggest that high levels of testosterone may increase sensitivity to consequences. Second, high levels of testosterone have been associated with reduced concern about punishment (e.g., van Honk et al., 2004), which suggest that high levels of testosterone may reduce sensitivity to moral norms. Finally, high levels of testosterone have been associated with stronger approach tendencies in the presence of threat (e.g., Enter et al., 2014), which suggest that high levels of testosterone may reduce general preference for inaction versus action. Testing effects of a double-blind intranasal administration of either testosterone or placebo in a sample of American undergraduate students, Brannon et al. (2019) found no evidence for either of the three hypotheses. Instead, they found that participants in the testosterone group showed stronger (rather than weaker) sensitivity to moral norms in moral decisions than participants in the placebo group. Interestingly, endogenous testosterone levels measured at baseline showed the opposite pattern, in that higher levels of endogenous testosterone were associated with weaker (rather than stronger) sensitivity to moral norms in moral decisions—consistent with the findings of Carney and Mason (2010) with the trolley problem. Together, these results cast doubts on the assumption that associations between endogenous testosterone levels and preference for utilitarian over deontological judgments reflect a causal effect of testosterone. Because experimentally manipulated testosterone levels showed a pattern that directly contradicts this assumption, it seems more likely that the obtained association between endogenous testosterone and moral-dilemma responses is driven by another variable that happens to be associated with both high levels of endogenous testosterone and low sensitivity to moral norms (e.g., psychopathy). If anything, the findings of Brannon et al. (2019) suggest that testosterone has the opposite causal effect by increasing (rather than decreasing) sensitivity to moral norms (for related findings, see Boksem et al., 2013; Eisenegger et al., 2010; Wibral et al., 2012).

### Action Orientations

By explicitly addressing the role of general action preferences, the CNI model suggests a mechanism by which broader differences in behavioral activation and behavioral inhibition may influence responses in moral dilemmas (see Carver & White, 1994; Lucas & Galinsky, 2015). Consistent with this idea, some trolleyology studies found that preference for utilitarian over deontological judgments is positively associated with individual differences in the behavioral activation system (BAS; e.g., Moore et al., 2011) and negatively associated with individual differences in the behavioral inhibition system (BIS; e.g., Van den Bos et al., 2011). However, across trolleyology studies, the results are rather mixed and inconclusive (see Gawronski et al., 2023). A similarly mixed pattern emerged in a series of four CNI model studies with American MTurk workers (Körner et al., 2020), which did not find any reproducible associations of BIS and BAS with the three CNI model parameters regardless of whether the responses involved moral judgments or moral decisions.

More consistent evidence for effects of action orientations was obtained by Cornwell and Bella (2022) who investigated the two regulatory modes of locomotion and assessment (see Kruglanski et al., 2000). While locomotion refers to the motivation to initiate and sustain smooth movement, assessment refers to the motivation to engage in critical evaluation and reflection. Across two studies—one correlated with American military cadets and one preregistered experimental with American MTurk workers—Cornwell and Bella (2022) found that a stronger locomotion mode was associated with stronger sensitivity to moral norms in moral judgments. According to the authors, adherence to norms and rules facilitates the initiation and sustainment of smooth movement from one state to another, thereby supporting salient goals under locomotion mode.

### Political Orientation

A common assumption in the broader morality literature is that liberals and conservatives have fundamentally different moral intuitions (e.g., Graham et al., 2009), which can contribute to affective polarization among political groups (see Finkel et al., 2020). Although moral-dilemma research primarily focuses on concerns about care and harm, prior evidence in the trolleyology paradigm is consistent with this concern, suggesting that liberals show a greater preference for utilitarian over deontological judgments than conservatives (e.g., Chan, 2019; Hannikainen et al., 2017; Piazza & Landy, 2013; Piazza & Sousa, 2014; O. A. Young et al., 2013). From the perspective of the CNI model, the obtained association between political orientation and moral-dilemma responses may reflect (a) stronger sensitivity to consequences among liberals (see Piazza & Sousa, 2014), (b) stronger sensitivity to moral norms among conservatives (see O. A. Young et al., 2013), or (c) greater aversion to actions interfering with current states of affairs among conservatives (i.e., status quo bias; see Samuelson & Zeckhauser, 1988). Across three studies with American Prolific workers, British Prolific workers, and American CloudResearch workers, respectively, Luke and Gawronski (2021a) found that liberals showed stronger sensitivity to consequences in moral judgments than conservatives. There were no differences between liberals and conservatives in terms of sensitivity to moral norms and general preference for inaction versus action. Together, these results suggest that moral disagreements between liberals and conservatives may arise because liberals are more concerned about the greater good than conservatives (see Piazza & Sousa, 2014). Yet, liberals and conservatives seem to be equally concerned about violations of moral norms, and they seem to have similar perceptions regarding the asymmetry between harm caused via action and harm caused via inaction. However, although the obtained difference in sensitivity to consequences was replicated across three studies with samples from two different countries, the association between liberal (vs. conservative) political orientation and sensitivity to consequences was rather small with correlations between *r* = .12 and *r* = .13.

### Basic Personality Traits

Different from the rather weak associations with political orientation, the three factors underlying moral-dilemma responses tend to show rather strong associations with basic personality traits. Exploring associations with the Big Five personality traits (John et al., 2008) in a sample of American MTurk workers, Luke and Gawronski (2022b) found that higher levels of openness were associated with stronger sensitivity to consequences, stronger sensitivity to moral norms, and stronger general preference for inaction versus action in moral judgments. In addition, extraversion showed a positive association with sensitivity to consequences, and agreeableness showed a positive association with sensitivity to moral norms. These findings replicated in a preregistered follow-up study with American MTurk workers (Luke & Gawronski, 2022a, Study 2). However, a preregistered follow-up study with American undergraduate students failed to replicate all associations except for the positive association between agreeableness and sensitivity to moral norms (Luke & Gawronski, 2022a, Study 1). A direct comparison between the three studies suggests that the failed replications in the undergraduate student sample are most likely due to lower internal consistencies of the CNI model parameters and restricted variance in several of the involved measures.

Two studies by Kroneisen and Heck (2020) with German participants recruited via internet communities investigated associations between the three factors underlying moral-dilemma responses and a subset of the HEXACO traits (Ashton & Lee, 2007). In a selective analysis of six out of the 18 possible associations between CNI model parameters and HEXACO traits, the authors found that emotionality was positively associated with sensitivity to consequences and general preference for inaction versus action in moral judgments, while honesty-humility was positively associated with sensitivity to moral norms. Conscientiousness was not significantly associated with any of the three CNI model parameters. The association between honesty-humility and sensitivity to moral norms was replicated for moral decisions in two preregistered studies with American Prolific workers, but these studies did not include measures of the other five HEXACO traits (Ng et al., 2022). Ignoring the failed replication study with undergraduate students (Luke & Gawronski, 2022a, Study 1), the significant associations with basic personality traits were in the range of |*r*| = .18 to |*r*| = .46 and were thus considerably larger than the small associations with political orientation. Together, these results suggest that basic personality traits can contribute to disagreements about the right course of action in moral dilemmas and that they play a more substantial role for such disagreements than political orientations.

### Religiosity

**Figure 18.**
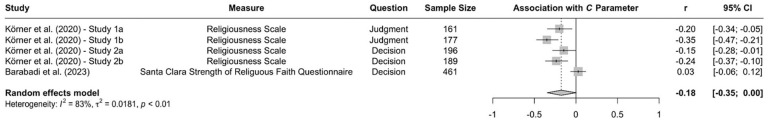
Associations of Religiosity With Sensitivity to Consequences. *Note.* Effect sizes are depicted as Pearson correlations. Higher correlations reflect a positive association between religiosity and a given parameter score. Error bars depict 95% confidence intervals.

**Figure 19.**
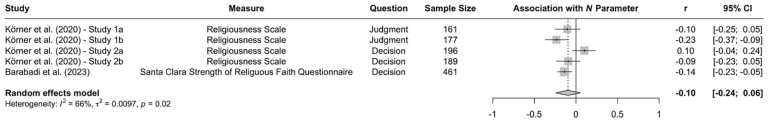
Associations of Religiosity With Sensitivity to Moral Norms. *Note.* Effect sizes are depicted as Pearson correlations. Higher correlations reflect a positive association between religiosity and a given parameter score. Error bars depict 95% confidence intervals.

**Figure 20.**
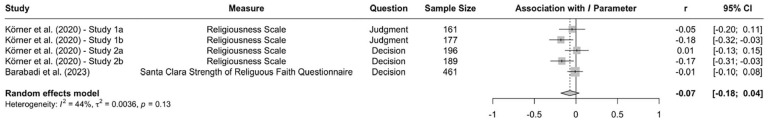
Associations of Religiosity With General Preference for Inaction Versus Action. *Note.* Effect sizes are depicted as Pearson correlations. Higher correlations reflect a positive association between religiosity and a given parameter score. Error bars depict 95% confidence intervals.

Research using the trolleyology paradigm has found a negative association between religiosity and preference for utilitarian over deontological judgments, which has been interpreted as indicating a greater concern about norm violations among religious individuals (e.g., Szekely et al., 2015). However, research using the CNI model suggests a different conclusion. Across four studies with American MTurk workers, Körner et al. (2020) found a negative association between religiosity and sensitivity to consequences, suggesting that religious individuals are less concerned about the greater good than non-religious individuals. Religiosity showed no reliable associations with sensitivity to moral norms and general preference for inaction versus action. This pattern of results emerged for both moral judgments and moral decisions. These findings speak against the assumption that the negative association between religiosity and preference for utilitarian over deontological judgments is driven by greater concerns about norm violations among religious individuals (see also Hofmann et al., 2014). If anything, a study with Iranian college students suggests the opposite, in that religiosity was negatively (not positively) associated with sensitivity to moral norms in moral judgments (Barabadi et al., 2023). When the available data are submitted to a meta-analysis (see Figures 18–20), religiosity showed a small negative association with the *C* parameter, but this association failed to reach the conventional level of statistical significance (*r* = −.177, 95% CI [−.347, .004], *t* = −2.71, *p* = .053). Religiosity showed no significant associations with sensitivity to moral norms (*r* = −.095, 95% CI [−.242, .056], *t* = −1.75, *p* = .154) and general preference for inaction versus action (*r* = −.072, 95% CI [−.182, .040], *t* = −1.79, *p* = .147).

### Gender Differences

Prior research using the trolleyology paradigm suggests that men show a stronger preference for utilitarian over deontological judgments than women (e.g., Fumagalli et al., 2010; see also Friesdorf et al., 2015). Two studies with American MTurk workers aimed to clarify why men and women differ in their responses to moral dilemmas (Gawronski et al., 2017, Experiments 1a and 1b).^10^ The two studies yielded reliable effects on the *N* and *I* parameters, in that women showed stronger sensitivity to moral norms and stronger general preference for inaction versus action in moral judgments than men. Effects on the *C* parameter were inconsistent across the two studies, in that women showed stronger sensitivity to consequences than men in one study, while the other study revealed no gender differences on the *C* parameter.

**Figure 21.**
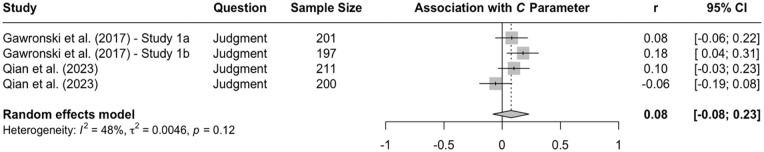
Gender Differences in Sensitivity to Consequences. *Note.* Effect sizes are depicted as Pearson correlations. Higher correlations reflect higher parameter scores among women than among men. Error bars depict 95% confidence intervals.

**Figure 22.**
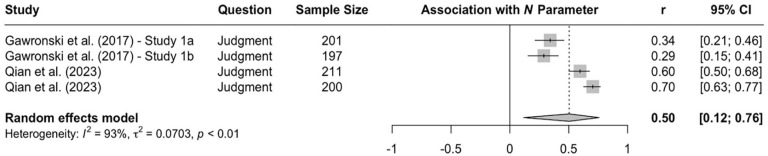
Gender Differences in Sensitivity to Moral Norms. *Note.* Effect sizes are depicted as Pearson correlations. Higher correlations reflect higher parameter scores among women than among men. Error bars depict 95% confidence intervals.

**Figure 23.**
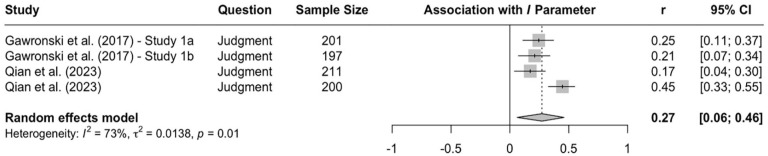
Gender Differences in General Preference for Inaction Versus Action. *Note.* Effect sizes are depicted as Pearson correlations. Higher correlations reflect higher parameter scores among women than among men. Error bars depict 95% confidence intervals.

Qian et al. (2023) replicated the gender difference in sensitivity to moral norms in a sample of Chinese university students and a sample of Japanese online workers on the crowdsourcing platform Questant. The gender difference in general preference for inaction versus action replicated only in the Chinese sample, but not in the Japanese sample. Sensitivity to consequences did not show significant gender differences in either sample. When the available data are submitted to a meta-analysis (see Figures 21–23), women show significantly stronger sensitivity to moral norms (*r* = .504, 95% CI [.117, .758], *t* = 4.04, *p* = .027) and significantly stronger general preference for inaction versus action (*r* = .272, 95% CI [.061, .460], *t* = 4.07, *p* = .027) than men. There was no significant gender difference in sensitivity to consequences (*r* = .077, 95% CI [−.080, .230], *t* = 1.56, *p* = .216).

### Cultural Differences

Although the trolleyology paradigm has been used in studies with participants from various countries around the globe, studies that directly investigated cultural differences in moral-dilemma responses are scarce. One notable exception is a study by Sorokowski et al. (2020), which found that indigenous participants from the remote Yalimo valley in Papua (Indonesia) showed a weaker preference for utilitarian over deontological judgments than participants from Canada. Another exception is a multi-lab study by Bago et al. (2022), which investigated the effect of physical contact on moral-dilemma responses in 45 countries from all inhabited continents. Replicating the findings of earlier studies with participants from the United States (see Greene, 2008), Bago et al. found stronger preferences for utilitarian over deontological judgments in the switch dilemma than in the footbridge dilemma in all 45 countries.

To gain deeper insights into the roots of potential cultural differences in moral-dilemma responses, a study by Speckmann et al. (2024) used the CNI model to compare moral judgments in participants from the United States (American MTurk workers) and Japan (Japanese Lancers workers). Based on prior work on cross-cultural differences in self-construal (Markus & Kitayama, 1991), the authors hypothesized that interdependent self-construals are associated with stronger sensitivity to consequences for the greater good, stronger sensitivity to moral norms, and stronger general preference for inaction versus action, whereas independent self-construals should show the opposite relations. Thus, to the extent that interdependent self-construals are more common in Japan and independent self-construals are more common in the United States (Markus & Kitayama, 1991), participants from Japan should show higher scores on all three CNI model parameters than participants from the United States. Consistent with the authors’ preregistered hypothesis for the *I* parameter, participants from Japan showed significantly stronger general preference for inaction versus action than participants from the United States. Yet, counter to the authors’ preregistered hypothesis for the *N* parameter, there was no significant difference for sensitivity to moral norms. For the *C* parameter, the authors obtained a significant effect that was opposite to their preregistered hypothesis, in that participants from Japan showed significantly weaker sensitivity to consequences than participants from the United States. Further analyses revealed that, although measured levels of independent self-construal were higher among participants from the United States than among participants from Japan, participants from the United States also showed significantly higher levels of interdependent self-construal than participants from Japan. Moreover, measured levels of independent self-construal showed a significant positive association with sensitivity to consequences, which may explain the unexpected country-level difference on the *C* parameter. Independent self-construal was not significantly related to sensitivity to moral norms and general preference for inaction versus action. Measured levels of interdependent self-construal were not significantly related to any of the three CNI parameters. Thus, although the findings of Speckmann et al. (2024) provide tentative evidence for country-level differences in responses to moral dilemmas, there is no support for the hypothesized role of self-construals.

Comparing existing data from American MTurk workers (Gawronski et al., 2017, Experiments 1a) to newly collected data from Chinese university students and Japanese online workers on the crowdsourcing platform Questant, Qian et al. (2023) found the same country-level difference on the *I* parameter, in that Japanese participants showed stronger general preference for inaction versus action in moral judgments than American participants. In terms of general action preferences, Chinese participants were more similar to American than Japanese participants, in that Japanese participants showed stronger general preference for inaction versus action than both American and Chinese participants, with American and Chinese participants not differing from each other. Different from the null effect on the *N* parameter in the study by Speckmann et al. (2024), American participants showed significantly stronger sensitivity to moral norms than both Chinese and Japanese participants. Sensitivity to consequences did not show reliable cultural differences in the study by Qian et al. (2023). Based on the available evidence, it seems fair to conclude that Japanese show stronger general preference for inaction versus action than American participants, but data of Qian et al. (2023) on Chinese participants and data of Speckmann et al. (2024) on measured self-construals suggest that this difference is driven by factors that are unrelated to the distinction between independent and interdependent self-construals. With the inconsistent effects on the *C* and *N* parameters, the available evidence does not permit strong conclusions about cultural differences in sensitivity to consequences and sensitivity to moral norms.

### Foreign-Language Use

In moral-dilemma studies investigating cross-cultural differences, the dilemmas are typically presented in participants’ native language, raising the question of how foreign-language use may influence moral-dilemma responses. In several studies using the trolleyology paradigm, participants showed a stronger preference for utilitarian over deontological judgments when they read the dilemmas in a foreign language as opposed to their native language (e.g., Costa et al., 2014; Geipel et al., 2015). This phenomenon has been called the moral foreign-language effect (MFLE). A meta-analysis on the MFLE (Stankovic et al., 2022) found a significant meta-analytic MFLE only for personal dilemmas that involve direct contact with the target of the described action (e.g., footbridge dilemma), but not for impersonal dilemmas that do not involve direct contact with the target (e.g., switch dilemma). Yet even the significant MFLE for personal dilemmas was very small with an effect size that fell below the conventional benchmark for a small effect.^11^

**Figure 24.**
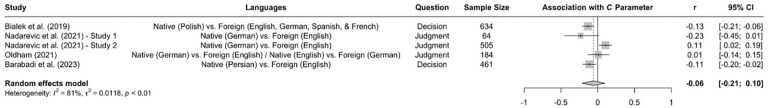
Effects of Foreign-Language Use on Sensitivity to Consequences. *Note.* Effect sizes are depicted as Pearson correlations. Higher correlations reflect higher parameter scores in the foreign-language condition than in the native-language condition. Error bars depict 95% confidence intervals.

**Figure 25.**
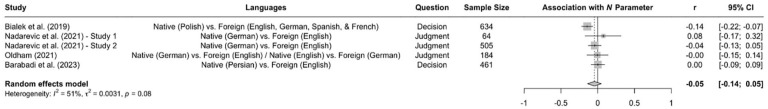
Effects of Foreign-Language Use on Sensitivity to Moral Norms. *Note.* Effect sizes are depicted as Pearson correlations. Higher correlations reflect higher parameter scores in the foreign-language condition than in the native-language condition. Error bars depict 95% confidence intervals.

**Figure 26.**
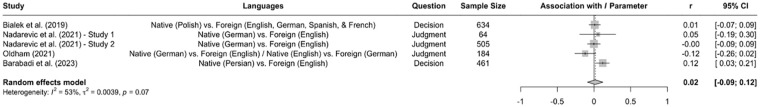
Effects of Foreign-Language Use on General Preference for Inaction Versus Action. *Note.* Effect sizes are depicted as Pearson correlations. Higher correlations reflect higher parameter scores in the foreign-language condition than in the native-language condition. Error bars depict 95% confidence intervals.

From the perspective of the CNI model, there are two potential explanations for this meta-analytic outcome. On the one hand, it is possible that effects of foreign-language use on moral-dilemma responses are rather small and unreliable (see Nadarevic et al., 2021). On the other hand, foreign-language use might influence moral-dilemma responses via multiple distinct factors, and these influences might compensate each other in the trolleyology paradigm where they cannot be delineated (see Białek et al., 2019). The currently available evidence supports the former conclusion. CNI model studies on the effects of foreign-language use include one study with 634 Polish bilinguals who read the dilemmas in either their native language or in English, Spanish, German, or French (Białek et al., 2019); two studies with German bilinguals who read the dilemmas in either their native language or English (Nadarevic et al., 2021); one study with German and English bilinguals who read the dilemmas in either German or English (Oldham, 2022); and one study with Iranian bilinguals who read the dilemmas in either Persian or English (Barabadi et al., 2023). A meta-analysis on the data from these studies did not reveal any significant effects of foreign-language use on any of the three CNI model parameters (see Figures 24–26). The meta-analytic correlations were close to zero for sensitivity to consequences (*r* = −.059, 95% CI [−.213, .098], *t* = −1.05, *p* = .353), sensitivity to moral norms (*r* = −.046, 95% CI [−.139, .048], *t* = −1.34, *p* = .250), and general preference for inaction versus action (*r* = .017, 95% CI [−.089, .122], *t* = 0.43, *p* = .688). Together with the very small meta-analytic MFLE in the trolleyology paradigm (Stankovic et al., 2022), these findings raise questions about the extent to which moral-dilemma responses are influenced by foreign-language use.

### Uncertainty

The scenarios in trolleyology research are typically phrased such that there is 100% certainty about the described outcomes. For example, in the footbridge dilemma, participants are supposed to be convinced that pushing the man off the bridge would definitely stop the trolley and save the lives of the five people and that the trolley would definitely kill the five people if the man is not pushed off the bridge. It is an open question if participants agree with these premises (Bennis et al., 2010), and for dilemmas in real-world contexts, the premise of 100% certainty about outcomes seems rather implausible. These issues raise the question of how uncertainty about outcomes influences moral-dilemma responses.

Addressing this question, a trolleyology study by Kortenkamp and Moore (2014) found that participants showed a weaker preference for utilitarian over deontological judgments when the dilemma outcomes were described with the probabilistic verb “might” (i.e., low certainty) than when the dilemma outcomes were described with the deterministic verb “will” (i.e., high certainty). Theoretically, the finding by Kortenkamp and Moore (2014) could be driven by (1) discounting of cost-benefit ratios under uncertainty (see Cherry & Fraedrich, 2002), (2) enhanced reliance on rules as a means to reduce uncertainty (see J. R. Smith et al., 2007), or (3) enhanced concern with potential losses under uncertainty (see Yeung et al., 2022). From the perspective of the CNI model, the first mechanism should be reflected in a weaker sensitivity to consequences under uncertainty; the second mechanism should be reflected in a stronger sensitivity to moral norms under uncertainty; and the third mechanism should be reflected in a stronger general preference for inaction versus action under uncertainty.

Results from four preregistered experiments by Ng et al. (2023) with Prolific workers from the United Kingdom support the first hypothesis, in that participants showed a weaker sensitivity to consequences when the dilemma outcomes were described with the probabilistic verb “might” (i.e., low certainty) than when the dilemma outcomes were described with the deterministic verb “will” (i.e., high certainty). This effect emerged regardless of whether the moral-dilemma question involved moral judgments or moral decisions. There were no reliable effects of outcome uncertainty on sensitivity to moral norms and general preference for inaction versus action. These results suggest that uncertainty about outcomes influences moral-dilemma responses via discounting of cost-benefit ratios.

### Perceived Intent

When people make moral judgments about the choices of someone else, condemnation of harm is strongly influenced by perceptions of intent (Malle, 2021). In trolleyology research, perceived intent seems relevant for the finding that participants typically show a stronger preference for utilitarian over deontological judgments in the switch dilemma than in the footbridge dilemma (Bago et al., 2022). Although this finding has been attributed to the differential involvement of physical contact in the two scenarios (Greene, 2008), sacrificial harm can be regarded as an intended means in the footbridge dilemma but as an unintended side-effect in the switch dilemma (Greene et al., 2009). To disentangle effects of intent and personal force applied to the victim, Greene et al. (2009) compared moral-dilemma responses across various modified versions of the switch and footbridge dilemmas, showing that harmful actions were judged least acceptable when sacrificial harm was intended and the result of personal force applied to the victim.

To gain deeper insights into the effects of perceived intent on moral-dilemma responses, Halliwell (2021) conducted a study with Australian university students in which the perspective of the agent in the dilemmas was changed from second person (e.g., You are the director of a hospital in a developing country) to third person (e.g., Chris is the director of a hospital in a developing country). In addition to the basic scenarios, participants received information about the actor’s intentions. For half of the participants, the described intentions were prosocial (good-intention condition); for the remaining half, the described intentions were selfish (bad-intention condition). Participants in both conditions were asked if it is appropriate for the agent to perform the described action. Based on prior research on the effects of perceived intent, Halliwell (2021) tested the hypotheses that sensitivity to consequences, sensitivity to moral norms, and general preference for inaction versus action would all be more pronounced when the actor has bad intentions than when the actor has good intentions. The results confirmed the author’s hypothesis for the *I* parameter, in that general preference for inaction versus action was significantly stronger when the agent had bad intentions than when the agent had good intentions. Put differently, participants generally opposed actions of agents with bad (vs. good) intentions regardless of the specific situation. Counter to the author’s predictions for the *C* and *N* parameters, there were no significant effects on sensitivity to consequences and sensitivity to moral norms. Although the approaches used by Greene et al. (2009) and Halliwell (2021) were quite different, these results suggest that perceived intent may influence moral-dilemma responses via general action preferences rather than via sensitivity to consequences or sensitivity to moral norms.

### Alcohol

A somewhat puzzling finding in trolleyology research is that higher levels of blood alcohol were found to be associated with a stronger preference for utilitarian over deontological judgments (Duke & Bègue, 2015). This phenomenon, which has been called the drunk-utilitarian effect, conflicts with the assumptions that (a) utilitarian judgments require inhibitory control of automatic emotional responses to the idea of causing harm and (b) alcohol impairs inhibitory control. In conjunction, these assumptions suggest that alcohol should reduce (not increase) preference for utilitarian over deontological judgments in the trolley problem.

From the perspective of the CNI model, two more plausible explanations of the drunk-utilitarian effect are that (a) alcohol reduces sensitivity to moral norms or (b) alcohol reduces general action aversion. To test these assumptions, Paruzel-Czachura et al. (2023) randomly assigned Polish participants from the local community to one of three conditions involving consumption of a drink that contained (a) 1.6 grams of alcohol at 40% strength for each 1 kg of the participant’s body weight (alcohol group), (b) only juice but no alcohol (no-alcohol group), or (c) only juice and no alcohol but was sprayed with alcohol to create the impression of alcohol consumption (placebo group). Participants in both the alcohol and the placebo groups (but not the no-alcohol group) were told that the drink contained alcohol. After a 51-minute absorption period, participants made moral decisions on the battery of moral dilemmas for research using the CNI model by Körner et al. (2020). Participants also responded to the switch and footbridge variants of the trolley problem. Although Paruzel-Czachura et al. (2023) used a substantially larger sample and higher doses of alcohol than prior studies, alcohol had no significant effect on trolley-dilemma responses. There was also no significant effect of alcohol on any of the three CNI model parameters. These results suggest that alcohol itself may have no causal effect on moral-dilemma responses and that prior correlational evidence for the drunk-utilitarian effect may be driven by a different variable that is associated with both alcohol consumption and moral-dilemma responses. For example, among the patrons of French bars recruited for the original study of Duke and Bègue (2015), those who are indifferent about potential harm to themselves caused by excessive alcohol consumption may also care less about harm in the trolley problem. If that is the case, the observed correlation may be driven by a general disposition to not care about harm instead of reflecting a causal effect of alcohol.

### Internet Addiction

Based on prior research suggesting that internet addiction is associated with impaired impulse control and risky decision-making (Ko et al., 2010), a study with Chinese participants from the local community investigated whether internet addiction is associated with systematic differences in moral-dilemma responses (Lu et al., 2022). To this end, the authors used the Internet Addiction Test of K. S. Young (1998) to preselect participants with either high scores (>50; addiction group) or low scores (<40; control group) on the test. Participants were also screened for nicotine dependence and alcohol use disorder to control for other addictions besides internet addiction. Participants in both groups were presented with moral dilemmas for research using the CNI model by Gawronski et al. (2017) and asked to indicate whether they would perform the described actions. Analyses revealed a significant group difference on the *N* parameter, indicating that participants in the internet-addiction group showed lower sensitivity to moral norms than participants in the control group. There were no significant effects on the *C* and *I* parameters. These results are consistent with the authors’ hypothesis that individuals suffering from internet addiction have a stronger propensity to make utilitarian judgments. However, the findings with the CNI model indicate that this propensity has little to do with greater concerns about outcomes, which should be reflected in a positive association between internet addiction and sensitivity to consequences. It also seems unlikely that the stronger propensity to make utilitarian judgments among individuals with internet addiction is rooted in impaired impulse control or greater willingness to make risky decisions, which should be reflected in a negative association between internet addiction and general preference for inaction versus action. A more plausible explanation, suggested by Lu et al. (2022), is that internet addiction influences moral-dilemma responses via reduced empathic concern resulting from impaired emotional processing. However, based on the available data, this interpretation remains speculative, requiring future research on the processes underlying effects of internet addiction of moral-dilemma responses.

### Behavior Prediction

Expanding on concerns about lack of realism and implausible assumptions in trolleyology scenarios (e.g., Bauman et al., 2014; Körner et al., 2019), some researchers argued that hypothetical responses in sacrificial dilemmas are ill-suited for understanding actual moral decisions in real-world contexts (e.g., Bostyn et al., 2018). Consistent with these concerns, the available evidence for predictive associations between responses in hypothetical trolleyology scenarios and instances of actual moral behavior is rather mixed (for a review, see Ng et al., 2022). However, in addition to the conflation of the three factors underlying moral-dilemma responses, a major limitation of prior work on this question is that many studies relied on single observations both to measure moral-dilemma responses and to measure instances of actual moral behavior. To control for the large proportions of measurement error in single-item measures (see Dang et al., 2020), a registered report by Ng et al. (2022) investigated associations between the three CNI model parameters and dishonest behavior using multiple observations for both predictors and outcomes. To measure dishonest behavior, American Prolific workers completed a simulated coin-flip task in which they were rewarded for each correctly predicted outcome (see Greene & Paxton, 2009; Shalvi & De Dreu, 2014). The task was designed such that participants could increase their reward by lying about their performance on each trial of the task. With a total of 70 prediction trials and two potential outcomes (i.e., heads vs. tails), overall accuracy scores exceeding 35 correct predictions are increasingly improbable and suggestive of dishonest reporting. In a first study, participants were told that the three participants with the best performance on the task would each receive a bonus payment of $50 in addition to their compensation for participating in the study (i.e., dishonest behavior serves personal monetary gain). In a second study, participants were told that the researchers will donate 10¢ to a charity of their choice for every correct prediction on the coin-toss prediction task (i.e., dishonest behavior serves the greater good).

The results provided support for the predicted negative association between sensitivity to moral norms and dishonest behavior irrespective of whether dishonest behavior served personal monetary gain or the greater good. There was no support for the predicted positive association between sensitivity to consequences and dishonest behavior for the greater good. In addition to providing nuanced evidence for associations between specific factors underlying moral-dilemma responses and instances of actual moral behavior, these findings suggest that specific factors underlying moral-dilemma responses are linked to other morally relevant behaviors via broader underlying moral dispositions.

### Summary

Table 3 provides a summary of the reviewed findings. Broken down by the three CNI model parameters, the available evidence suggests that sensitivity to consequences increases with greater levels of cognitive reflection, self-importance of moral identity internalization, liberal political ideology, extraversion, openness, and emotionality. Conversely, sensitivity to consequences decreases with greater levels of psychopathy and outcome uncertainty. Sensitivity to moral norms increases as a function of cognitive reflection, empathic concern, self-importance of moral identity internalization, incidental guilt, structural power, exogenous testosterone, a motivational state of locomotion, agreeableness, openness, and honesty-humility. Conversely, sensitivity to moral norms decreases as a function of psychopathy, permissive attitudes toward sacrificial harm, incidental happiness, incidental envy, psychological power, endogenous testosterone, and internet addiction. Moreover, sensitivity to moral norms is stronger for moral judgments than for moral decisions and among women than among men. Sensitivity to moral norms is also negatively associated with dishonest behavior regardless of whether it serves personal gains or the greater good. Finally, general preference for inaction versus action increases with increasing levels of stress, openness, and perceived negative intentions, and it decreases with increasing levels of psychopathy, permissive attitudes toward sacrificial harm, and impartial concern for the wellbeing of everyone. General preference for inaction versus action is also stronger for moral decisions than for moral judgments, among women than among men, and among Japanese than among U.S. Americans. As we discussed in the preceding individual sections, many of these findings resolve ambiguities in the interpretation of findings obtained with the trolleyology paradigm. Moreover, a subset of the reviewed findings has important implications for extant theories about the processes underlying moral-dilemma responses, which we discuss in the following section.

**Table 3.** Overview of Empirical Findings With the CNI Model.

Variable	*C*	*N*	*I*
Judgments vs. Decisions^a,b^		-	+
Cognitive reflection^a^	+	+	
Psychopathy^a^	-	-	-
Empathic concern		+	
Instrumental harm (OUS)^a^		-	-
Impartial beneficence (OUS)^a^			-
Moral identity	+	+	
Incidental emotions: Happiness		-	
Incidental emotions: Sadness			
Incidental emotions: Anger			
Incidental emotions: Envy		-	
Incidental emotions: Guilt		+	
Stress			+
Social power: Psychological		-	
Social power: Structural		+	
Testosterone: Exogenous		+	
Testosterone: Endogenous		-	
Behavioral activation (BAS)			
Behavioral inhibition (BIS)			
Regulatory mode: Locomotion		+	
Regulatory mode: Assessment			
Conservative (vs. Liberal) Ideology^c^	-		
Personality: Extraversion	+		
Personality: Agreeableness		+	
Personality: Conscientiousness			
Personality: Openness	+	+	+
Personality: Neuroticism			
Personality: Honesty-Humility		+	
Personality: Emotionality	+		
Gender^a,d^		+	+
Religiosity^a^			
Country: USA vs. Japan^e^			+
Foreign-language use^a^			
Uncertainty	-		
Perceived negative intention			+
Alcohol			
Internet addiction		-	
Dishonest behavior for personal gains		-	
Dishonest behavior for the greater good		-	

*Note. C* = sensitivity to consequences; *N* = sensitivity to moral norms; *I* = general preference for inaction versus action; plus signs (+) indicate positive associations; minus signs (-) indicate negative associations; no cell entries indicate inconclusive or absence of evidence for either a positive or a negative association. OUS = Oxford Utilitarianism Scale; BAS = Behavioral Activation Scale; BIS = Behavioral Inhibition Scale.

a Entries are based on meta-analytic data. ^b^High = decisions, low = judgments. ^c^High = conservative, low = liberal. ^d^High = female, low = male. ^e^High = Japan, low = United States.

## Theoretical Implications

Although several theories have been proposed to explain responses in moral dilemmas (e.g., D. J. Cohen & Ahn, 2016; Cushman, 2013; Greene, 2008; Holyoak & Powell, 2016), the most prominent mechanistic account is Greene’s (2008) dual-process model (DPM). Expanding on the assumptions of extant dual-system theories of judgment and decision-making (e.g., Kahneman, 2003; for a review, see Gawronski et al., in press), the DPM states that utilitarian judgments are the product of controlled cognitive analyses of costs and benefits, whereas deontological judgments are rooted in automatic emotional reactions to the idea of causing harm. Findings commonly cited in support of the DPM are that (a) contextual factors that undermine cognitive reflection (e.g., time pressure, cognitive load) interfere with utilitarian judgments in the trolleyology paradigm (e.g., Greene et al., 2008; Suter & Hertwig, 2011) and (b) people show a stronger preference for deontological judgments in the footbridge dilemma than in the switch dilemma, presumably because direct physical contact with the target in the footbridge dilemma elicits a stronger emotional reaction to the idea of causing harm (e.g., Greene, 2008). Other frequently cited evidence is that utilitarian judgments in the trolleyology paradigm are associated with activation in brain areas claimed to signify cognitive reasoning and executive control, while deontological judgments are associated with activation in brain areas claimed to signify emotional processing (e.g., Greene et al., 2001, 2004).

Although the CNI model and the DPM may seem to offer competing accounts of moral-dilemma responses, there is no a priori conflict between the two theories because they are concerned with different levels of analysis (see De Houwer, 2011; Gawronski & Bodenhausen, 2015). While the CNI model is a descriptive theory that quantifies patterns of responses at the behavioral level, the DPM is a mechanistic theory that aims to specify the mental processes underlying behavioral responses (see Gawronski et al., 2018). From this perspective, an important question is how the empirical findings obtained with the CNI model relate to the mechanistic assumptions of the DPM.

To answer this question, it is essential to first clarify how the distinction between utilitarian and deontological judgments maps onto the three parameters of the CNI model (Dale & Gawronski, 2023). The mapping is relatively simple for utilitarian judgments, in that utilitarian outcome maximization is directly captured by the CNI model’s *C* parameter, which reflects the tendency to support action when the benefits of action are greater than the costs and to support inaction when the benefits of action are smaller than the costs. A similar argument could be made for the mapping of deontological judgments and the CNI model’s *N* parameter, which reflects the tendency to support inaction when a proscriptive norm prohibits action and to support inaction when a prescriptive norm prescribes action. However, deontological judgments could also be mapped onto the pattern of general action aversion captured by the CNI model’s *I* parameter given that adherence to the deontological norm “first, do no harm” can lead to a general preference to inaction over action (Baron & Goodwin, 2020). Indeed, when viewed from this angle, the response pattern captured by the *I* parameter seems much closer to the explanatory construct of negative emotional reactions to the idea of causing harm than the response pattern captured by the *N* parameter. While the *N* parameter captures the extent to which responses are shaped by the described proximal effects (i.e., support for inaction when action causes proximal harm and support for action when action prevents proximal harm; see second row in Figure 1), the *I* parameter captures the extent to which people show a general preference for inaction (vs. action) regardless of the proximal and distal effects of action (see third and fourth rows in Figure 1). Thus, when analyzing the relation between the DPM and empirical findings obtained with the CNI model, it is essential to consider both potential interpretations of deontological responding and the specific response patterns captured by the three CNI model parameters.

Among the variables that have been investigated in research using the CNI model (see Table 3), cognitive reflection is most closely related to the assumptions of the DPM. According to the DPM, utilitarian judgments are the product of controlled cognitive analyses of costs and benefits, whereas deontological judgments are rooted in automatic emotional reactions to the idea of causing harm. Hence, greater cognitive reflection should be positively associated with utilitarian judgments but unrelated to deontological judgments. These assumptions are consistent with the obtained meta-analytic association between cognitive reflection and the *C* parameter (see Figure 6), indicating that sensitivity to consequences increases as a function of cognitive reflection. Moreover, the lack of a significant association between cognitive reflection and the *I* parameter is consistent with the idea that general preference for inaction versus action arises from automatic emotional reactions to the idea of causing harm. However, the DPM is unable to account for the positive association between cognitive reflection and the *N* parameter (see Figure 7), which is larger than the positive association between cognitive reflection and the *C* parameter. Conceptually, the positive association between cognitive reflection and the *N* parameter indicates that cognitive reflection enhances support for inaction when action causes proximal harm and support for action when action prevents proximal harm. The DPM does not include any assumptions that could explain this effect. Because the *N* parameter explains a considerable portion of variance in the trolleyology paradigm that is not captured by the *C* and *I* parameters (see Figure 2), the DPM cannot be protected from this explanatory gap by dismissing the CNI model paradigm and using the trolleyology paradigm as the sole basis for evaluations of the DPM.

Another variable that seems highly relevant for evaluations of the DPM is the difference between moral judgments and moral decisions. Our meta-analytic review suggests that sensitivity to moral norms is stronger for moral judgments than for moral decisions, whereas general preference for inaction versus action is stronger for moral decisions than for moral judgments (see Figures 4–5). Considering that decisions about whether to perform a given action (i.e., moral decisions) are associated with greater personal involvement than abstract judgments about the acceptability of a given action (i.e., moral judgments), the obtained result for the *I* parameter is consistent with the DPM’s hypothesis about the role of automatic emotional reactions to the idea of causing harm. Specifically, one could argue that the greater personal involvement in moral decisions (vs. moral judgments) enhances automatic emotional reactions to the idea of causing harm, which in turn leads to stronger action aversion irrespective of the specific situation. However, the DPM has difficulties accounting for the obtained difference on the *N* parameter, which suggests that sensitivity to moral norms is weaker under conditions of strong personal involvement. Considering that the response patterns captured by the *N* and *I* parameters can both be described as instances of deontological responding (Dale & Gawronski, 2023), the DPM is unable to explain why stronger personal involvement increases one type of deontological responding (consistent with the DPM’s assumptions about the mechanisms underlying deontological responses) but decreases another type of deontological responding (inconsistent with the DPM’s assumptions about the mechanisms underlying deontological responses).

The DPM also faces difficulties in accounting for the effect of happiness, which has been found to reduce sensitivity to moral norms (Gawronski et al., 2018). Although this finding may seem consistent with the hypotheses that (a) deontological responses are rooted in negative emotional responses to the idea of causing harm and (b) happiness dampens negative emotional responses, it remains unclear how negative emotional reactions to the idea of causing harm could produce a preference for action when a prescriptive norm prescribes action (see Gawronski et al., 2018). To be sure, negative emotional reactions to the idea of causing harm may produce a preference for inaction when a proscriptive norm prohibits harmful action. However, such negative emotional reactions should increase action aversion regardless of whether the involved moral norm is proscriptive or prescriptive. From this perspective, the DPM would suggest that happiness should reduce general preference for inaction versus action, not sensitivity to moral norms. Thus, a more plausible interpretation of the obtained effect of happiness is that it influences moral-dilemma responses by dampening negative emotional reactions to the idea of violating moral norms (see Nichols & Mallon, 2006). Because the DPM explicitly rejects thoughts about moral norms as a driving force underlying deontological judgments, it is difficult to see how the DPM could be reconciled with such an interpretation.

In sum, although the CNI model and the DPM are concerned with different levels of analysis—and thus are not necessarily in conflict with each other—various findings obtained with the CNI model pose a challenge to the validity of the DPM. The most significant findings in this regard are that (a) cognitive reflection increases sensitivity to moral norms, (b) personal involvement reduces sensitivity to moral norms, and (c) happiness reduces sensitivity to moral norms. Because sensitivity to moral norms plays a major role in moral-dilemma responses (see Table 3), including those in the trolleyology paradigm (see Figure 2), these findings suggest that the DPM provides an incomplete, if not incorrect account, of the processes underlying moral-dilemma responses. Hence, future theoretical work is needed to obtain a mechanistic account of moral-dilemma responses that could replace the DPM. We will return to this point when we discuss open questions and new directions.

## Criticism

Although the CNI model has provided more nuanced insights into the underpinnings of moral-dilemma responses than the trolleyology paradigm, the model has also been the target of criticism. One concern raised by Baron and Goodwin (2020) is that participants tend to disagree with a classification of specific CNI model dilemmas by Gawronski et al. (2017) in terms of consequences and moral norms. According to Baron and Goodwin (2020), such disagreements raise questions about the construct validity of the *C* and *N* parameters as indicators of sensitivity to consequences and sensitivity to moral norms, respectively. The gist of the critique is that because participants perceive cost-benefit ratios and moral norms in the four dilemma variants in a manner that is different from the intended operationalizations of cost-benefit ratios and moral norms, the parameters capturing sensitivity to consequences and sensitivity to moral norms have low construct validity.

In a response to the critique of Baron and Goodwin (2020), Gawronski et al. (2020) argued that their concern is based on a misunderstanding of what the parameters of the CNI model are meant to capture, in that the critique conflates stimulus-response relations at the behavioral level with underlying processes at the mental level (see De Houwer, 2011; Gawronski & Bodenhausen, 2015). As explained in more detail by Gawronski et al. (2018), the CNI model is a descriptive theory that captures stimulus-response relations at the behavioral level; the model does not include any assumptions about the mental processes underlying particular patterns of stimulus-response relations. Thus, by using participants’ conscious thoughts about cost-benefit ratios and moral norms as a criterion for the classification of moral dilemmas, the critique of Baron and Goodwin (2020) conflates the two levels of analyses by assuming that the manipulation of consequences must influence responses via conscious thoughts about costs and benefits and that the manipulation of moral norms must influence responses via conscious thoughts about moral norms. The CNI model does not make any such assumptions.

In technical terms, the parameters of the CNI model are intended to capture patterns of judgments that merely conform to a rule irrespective of whether the rule is consciously considered in the generation of these judgments (i.e., rule-conforming judgments); they were never intended to capture judgments that arise from the conscious consideration of a particular rule (i.e., rule-following judgments). For the *C* parameter, the rule describing the measured stimulus-response relations at the behavioral level is support for action when the described benefits of action are greater than the costs and support for inaction when the described benefits of action are smaller than the costs (with costs and benefits referring to the described outcomes in the scenario rather than subjective construals of costs and benefits). For the *N* parameter, the rule describing the measured stimulus-response relations at the behavioral level is support for inaction when action causes proximal harm and support for action when action prevents proximal harm (with proximal harm referring to the immediate effect described in the scenario rather than subjective construals of effects). The distinction between rule-conforming and rule-following judgments is important because participants may show response patterns that conform to either of the two rules without engaging in conscious reasoning about cost-benefit ratios or moral norms pertaining to proximal harm. Thus, although the critique by Baron and Goodwin (2020) would be valid if the CNI model had been intended to capture rule-following judgments, it does not apply to the intended interpretation of the model parameters as capturing rule-conforming judgments (see Gawronski et al., 2020).

Although the argument of Baron and Goodwin (2020) is based on a misunderstanding of the CNI model, it is still an important question whether the manipulations of consequences and moral norms in the CNI model dilemmas are construct-valid at the behavioral level, and whether this is the case for each basic dilemma in its four variants (see Figure 1). To address this question, Gawronski et al. (2020) conducted an item-level analysis examining whether the response patterns in each set of four dilemma variants reliably reflect the manipulations of consequences and moral norms. Their analysis supported the construct validity of all sets of four dilemma variants, the only exception being a scenario called the abduction dilemma, which showed poor validity for the manipulation of moral norms. Based on these findings, Gawronski et al. (2020) recommended excluding the four variants of the abduction dilemma from future research with the CNI model. Together with the additional dilemmas developed by Körner et al. (2020), the available battery of dilemmas for research using the CNI model includes 11 basic scenarios in four variants (i.e., 44 dilemmas total).

Another concern pertains to the hierarchical relation of the three model parameters in the processing tree (see Figure 1), which is empirically arbitrary in the sense that any combinatorially possible arrangement of the three parameters will show the same goodness-of-fit for a given data set (see Gawronski et al., 2017). Yet, the numeric scores for the three parameters can differ across model specifications, raising the question of whether the results obtained with the standard model replicate with other model specifications. In line with this concern, Baron and Goodwin (2020) claimed that switching the position of *C* and *N* in the processing tree (i.e., NCI) leads to outcomes that are opposite to the ones with the original model (i.e., CNI). However, it is worth noting that the comparison of different model specifications by Baron and Goodwin (2020) was based on analyses with an entirely different model.^12^ It did not include the original model (i.e., CNI), nor did it include a version of the model in which the positions of *C* and *N* in the processing tree were switched (i.e., NCI). To examine the validity of the claims about contradictory outcomes by Baron and Goodwin (2020), Gawronski et al. (2020) re-analyzed all data from the original article by Gawronski et al. (2017) on the CNI model using an alternative NCI model structure. All original results replicated when the positions of *C* and *N* in the processing tree were switched. Thus, although it seems important to establish the reliability of a given finding across model specifications, there is no evidence for the claim by Baron and Goodwin (2020) that changes in model specifications can lead to opposite empirical outcomes. We return to this issue when we discuss alternative approaches.

Another concern raised by Baron and Goodwin (2020, 2021) is that the CNI model requires what they call “perverse responses” on dilemmas where consequences and moral norms support the same choice (see second and third columns in Figure 1). According to Baron and Goodwin (2020), participants show a “perverse response” when they favor action although consequences and moral norms would both support inaction, or when they favor inaction although consequences and moral norms would both support action. This argument includes a normative and a methodological aspect. The normative aspect pertains to the classification of moral-dilemma responses as “perverse.” We deem such a classification problematic because it invokes moral opinions held by scientists, and these opinions are deemed superior to the moral opinions reflected in participants’ judgments. In our view, psychological research on moral-dilemma responses should investigate their underpinnings, determinants, and consequences. Normative evaluations of participants’ responses as “perverse” are not only outside the realm of empirical psychology; they can also hinder scientific progress. For example, one important aspect that is ignored in the normative appraisal of Baron and Goodwin (2020) is that various factors can make people prefer inaction over action even when consequences and moral norms would suggest action (e.g., the tendency to perceive harm caused by action as more severe than harm caused by inaction; see Yeung et al., 2022). Conversely, some people may not care about morality and support action even when consequences and moral norms would suggest inaction (e.g., people high in psychopathy; see Lu et al., 2022; Luke & Gawronski, 2021b). Labeling either of these responses “perverse” does not provide any insights into why people respond in the way they do. If anything, it seems detrimental to this endeavor because it shifts the focus from empirical to normative issues.

Although we have concerns about the normative aspect of the argument by Baron and Goodwin (2020), the methodological aspect of their argument is valid insofar as the MPT approach underlying the CNI model would be unable to produce a converging solution if participants show a response pattern that either fully conforms to the response pattern captured by the *C* parameter (see first row in Figure 1) or fully conforms to the response pattern captured by the *N* parameter (see second row in Figure 1). In such cases, the maximum-likelihood approach underlying MPT modeling is unable to generate estimates for the respective other two parameters, which undermines convergence. Moreover, if participants show a mixed response pattern that can be fully described by a combination of *C* and *N*, estimates for the *I* parameter will be unreliable because there are no data points to quantify general preference for inaction versus action. Either of these cases is characterized by what Baron and Goodwin (2020) call a lack of “perverse responses” on dilemmas where consequences and moral norms support the same choice. The limits of the CNI model in generating reliable parameter estimates in these cases is rooted in the hierarchical structure of the model. We will discuss potential ways to address this limitation in the next section when we discuss alternative approaches.

Finally, some have argued that the CNI model ignores known psychological asymmetries between proscriptive and prescriptive norms (see Janoff-Bulman et al., 2009) by measuring adherence to both kinds of norms in a single parameter (e.g., McPhetres et al., 2018; Reynolds et al., 2019; see also Baron & Goodwin, 2020). This critique ignores the fact that the presumed asymmetry is captured by the difference between the *N* and *I* parameters. While the *N* parameter captures a symmetric pattern of responding that conforms to both proscriptive and prescriptive norms, the *I* parameter captures an asymmetric response pattern that conforms to proscriptive norms but conflicts with prescriptive norms. Hence, counter to the claim that the CNI model ignores the difference between the two kinds of norms, the model explicitly allows for the possibility of either symmetric or asymmetric effects, providing a computational tool to quantify both cases (see Gawronski et al., 2020).

However, there is also a methodological aspect of the proposed asymmetry between proscriptive and prescriptive norms that has the potential to distort parameter estimates of the CNI model. A central premise of MPT models is that a given parameter remains invariant across stimulus classes (Klauer et al., 2015). Applied to the CNI model’s *N* parameter, the assumption of invariance would be violated if the impact of proscriptive norms is stronger than the impact of prescriptive norms. Because violations of invariance cannot be tested in the original CNI model paradigm, it remains unclear whether findings obtained with the CNI model are compromised by invariance violations. To address this concern, Skovgaard-Olsen and Klauer (2023) used a modified moral-judgment task that included an option to skip dilemmas and applied an extended version of the CNI model with an additional parameter for skip responses. Using their modified paradigm, the authors found evidence for invariance violations in two studies with American MTurk workers, which led them to suggest that parameter estimates of the CNI model could be compromised. However, in evaluating this conclusion, it is important to consider several conceptual and methodological aspects of studies by Skovgaard-Olsen and Klauer (2023).

First, counter to the original concern that proscriptive norms have a stronger impact than prescriptive norms, Skovgaard-Olsen and Klauer (2023) found the opposite pattern. To account for the unexpected finding, the authors speculated that prescriptive norms might have a stronger impact than proscriptive norms when the former involve interference with harmful actions of someone else. However, this interpretation would suggest that not preventing someone else from causing harm (e.g., not preventing someone from killing a person) is perceived as a more severe norm violation than actively causing the same harm (e.g., actively killing a person), which directly contradicts extant evidence on omission bias (for a meta-analysis, see Yeung et al., 2022). More seriously, even if the post hoc interpretation of Skovgaard-Olsen and Klauer (2023) was correct, it would apply only to a small subset of CNI model dilemmas. It is not applicable to the majority of CNI model dilemmas where the prescriptive norm does not involve interference with harmful actions of someone else (see Körner et al., 2020). Yet, these dilemmas showed the same unexpected asymmetry in the studies by Skovgaard-Olsen and Klauer (2023), suggesting that prescriptive norms have a stronger impact than proscriptive norms. This issue raises the question of whether the inclusion of a skip option influenced responses in a manner that produced this rather implausible outcome.

Second, and directly related to the previous point, it is worth noting that violations of invariance cannot be tested in the original CNI paradigm and that any such tests require non-trivial modifications of the task—such as the inclusion of a skip option in the research of Skovgaard-Olsen and Klauer (2023). To the extent that these modifications influence responses in a systematic manner, it seems impossible to draw inferences about properties of the original task from results obtained with the modified task. After all, it is possible that the invariance violations obtained with the modified task were caused by the required modifications and that no invariance violations occur without such modifications. This issue becomes especially concerning considering the finding of Skovgaard-Olsen and Klauer (2023) that prescriptive norms had a stronger impact than proscriptive norms in their modified CNI paradigm—a rather puzzling finding that conflicts with a large body of research in the morality literature (Janoff-Bulman et al., 2009). Thus, although it is difficult to rule out invariance violations in the original CNI model paradigm, conceptual and methodological issues undermine the possibility of drawing inferences from the findings of Skovgaard-Olsen and Klauer (2023) about invariance violations in the original CNI model paradigm.

## Relation to Alternative Approaches

Beyond the CNI model, several other computational models have been proposed to disentangle multiple distinct factors underlying responses to moral dilemma (e.g., Conway & Gawronski, 2013; Hennig & Hütter, 2020; Liu & Liao, 2021; Skovgaard-Olsen & Klauer, 2023). In this section, we describe the available alternatives and their relations to the CNI model.

### Process Dissociation Model

**Figure 27.**
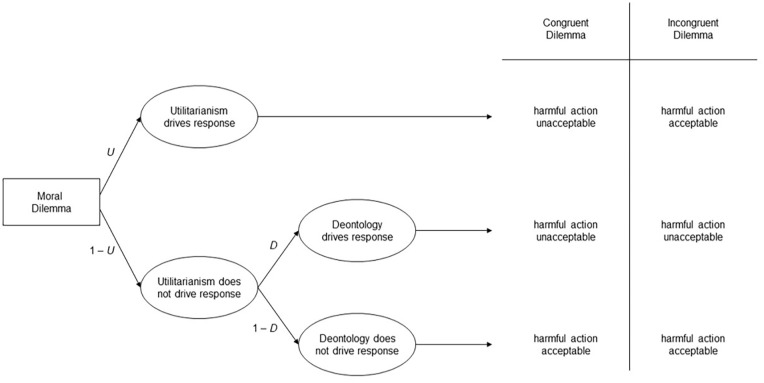
Processing Tree of the Process Dissociation Model of Moral-Dilemma Responses Predicting Judgments of Harmful Actions as Acceptable or Unacceptable as Function of Dilemma Type (Incongruent vs. Congruent). *Source.* Adapted from Conway and Gawronski (2013). Reprinted with permission from the American Psychological Association.

A precursor to the CNI model is the process dissociation (PD) model of Conway and Gawronski (2013), which utilizes a procedure from memory research (Jacoby, 1991) to resolve the non-independent measurement of utilitarian and deontological tendencies in the trolleyology paradigm. To this end, the PD model compares responses across dilemmas where utilitarianism and deontology would suggest either the same choice (i.e., congruent dilemmas) or different choices (i.e., incongruent dilemmas). Incongruent dilemmas are structurally equivalent to the trolley problem, in that they involve harmful actions that increase overall well-being. Congruent dilemmas involve harmful actions that do not increase overall wellbeing. Similar to the CNI model, the PD model can be depicted as a processing tree in which responses on congruent and incongruent dilemmas are linked to unique paths where either utilitarian or deontological tendencies drive responses (see Figure 27). Utilitarian tendencies are captured by the PD model’s *U* parameter, which reflects the extent to which participants support action on incongruent dilemmas and inaction on congruent dilemmas. Deontological tendencies are captured by the PD model’s *D* parameter, which reflects the extent to which participants support inaction (vs. action) on both incongruent and congruent dilemmas. Based on the paths in processing tree, one can derive two equations that include the two parameters as unknowns and the observed probabilities of supporting action (versus inaction) in two types of dilemmas as known numerical values. Because the PD model includes only two equations with two unknowns, parameter scores can be calculated directly via linear algebra. By providing separate scores for utilitarian and deontological tendencies, the PD model resolves the non-independent measurement of utilitarian and deontological judgments in the trolleyology paradigm, where endorsement of the utilitarian option necessarily involves a rejection of the deontological option, and vice versa.

The separation of the two tendencies in the PD model has provided more nuanced insights into the underpinnings of multiple ambiguous findings in the trolleyology paradigm (see Conway et al., 2018). However, a major disadvantage of the PD model is its exclusive focus on dilemmas where proscriptive norms prohibit action. Because the PD model does not consider cases where action is prescribed by prescriptive norms, the response pattern captured by the *D* parameter involves general support for inaction (vs. action) regardless of dilemma type (see second and third rows in Figure 27). As such, the *D* parameter confounds the norm-conforming response pattern captured by the CNI model’s *N* parameter with general action preferences captured by the CNI model’s *I* parameter (Gawronski et al., 2016). Yet, as shown in the current review, sensitivity to moral norms and general preference for inaction versus action are functionally and psychologically distinct, in that their corresponding parameters can be affected differently by the same variable (see Table 3). One example is the difference between moral judgments and moral decisions, in that sensitivity to moral norms is stronger for moral judgments than moral decisions, whereas general preference for inaction versus action is stronger for moral decisions than moral judgments (see Figures 4 and 5). Because the two effects compensate each other in the PD model’s *D* parameter, the PD model would falsely suggest that there is no systematic difference between moral judgments and moral decisions. These considerations suggest that the confound between sensitivity to moral norms and general action preferences in the PD model’s *D* parameter can lead to incorrect conclusions. Moreover, by not controlling for general response tendencies, scores for the PD model’s *U* parameter can be distorted as well (Hütter & Klauer, 2016). Thus, although the PD model’s *U* parameter and the CNI model’s *C* parameter are conceptually equivalent, the *C* parameter most likely provides a cleaner proxy of utilitarian outcome-sensitivity than the *U* parameter.

### proCNI Model

**Figure 28.**
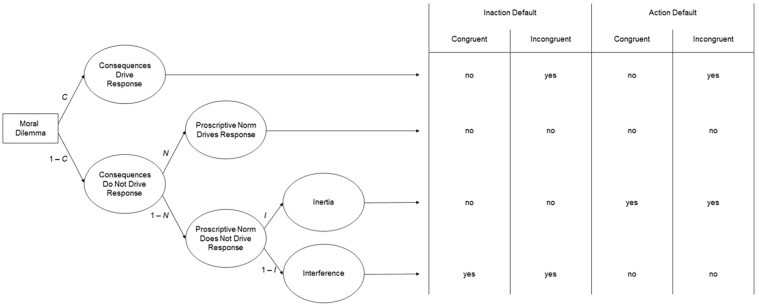
Processing Tree of the proCNI Model of Moral-Dilemma Responses Predicting Approval of Harmful Actions (Yes vs. No) as a Function of Dilemma Type (Congruent vs. Incongruent) and Defaults (Inaction vs. Action). *Source.* Adapted from Hennig and Hütter (2020). Reprinted with permission from the American Psychological Association.

Another computational model to disentangle multiple factors underlying moral-dilemma responses is the proCNI model of Hennig and Hütter (2020) (see Figure 28). Although the proCNI model may seem very similar to the CNI model, it is conceptually much closer to the PD model of Conway and Gawronski (2013). Similar to the PD model, the proCNI model exclusively focuses on dilemmas where proscriptive norms prohibit action (hence, the pro in proCNI). Moreover, while the incongruent proCNI dilemmas involve harmful actions that increase overall well-being (similar to the trolley problem), the congruent proCNI dilemmas involve harmful actions that do not increase overall well-being. The main difference to the PD model is that the proCNI model includes an additional parameter capturing inertia, which reflects the tendency to not change or interfere with a current state of affairs. Although inertia may seem closely related to general preference for inaction, the two are conceptually distinct because inertia can involve aversion to interference with either action or inaction. For example, if the default is inaction, aversion to interference with the default would result in inaction. However, if the default is action, aversion to interference would result in action. Thus, although the proCNI model uses the same parameter acronyms as the CNI model, the conceptual meaning of their *I* parameters is fundamentally different. While the *I* parameter of the CNI model captures general preference for inaction versus action, the *I* parameter of the proCNI model captures inertia, which may involve a preference for inaction when the default is inaction and a preference for action when the default is action.

Similar concerns apply to a potential equation of the proCNI model’s *N* parameter with the CNI model’s *N* parameter. Because the proCNI model focuses exclusively on dilemmas with proscriptive norms, the response pattern captured by the proCNI model’s *N* parameter involves general support for inaction (vs. action) regardless of dilemma type (see second row in Figure 28). As such, the proCNI model’s *N* parameter includes the same confound as the PD model’s *D* parameter, in that it conflates the norm-conforming response pattern captured by the CNI model’s *N* parameter with general action preferences captured by the CNI model’s *I* parameter. Thus, despite the use of the same acronym, the proCNI model’s *N* parameter is rather different from the CNI model’s *N* parameter, and conceptually much closer to the PD model’s *D* parameter. Together, these considerations suggest that findings obtained with the proCNI model (e.g., Hennig & Hütter, 2020, 2021) may not involve corresponding effects on the three CNI model parameters with the same acronym. For example, an effect on the proCNI model’s *N* parameter may be driven by general preference for inaction versus action, which should lead to an effect on the CNI model’s *I* parameter. Similarly, an effect on the proCNI model’s *I* parameter may not be detectable with the *I* parameter of the CNI model (and vice versa) because the two parameters are conceptually distinct. That being said, if future studies reveal reproducible effects on inertia that are distinct from effects on general action aversion, it might be worth developing an extended version of the CNI model that includes an additional parameter for inertia.

### CNIS Model

The CNIS model has been developed to address concerns about potential invariance violations in the CNI model (Skovgaard-Olsen & Klauer, 2023). A central premise of MPT models is that a given parameter remains invariant across stimulus types (Klauer et al., 2015). As noted earlier in this article, there are concerns that the invariance assumption is violated in the CNI model because proscriptive norms tend to be stronger than prescriptive norms (e.g., killing someone is perceived as a more severe norm violation than not saving someone’s life; see Janoff-Bulman et al., 2009). To address this concern, Skovgaard-Olsen and Klauer (2023) developed a modified moral-dilemma task in which participants are allowed to skip dilemmas when they are undecided about their choice. By adding an *S* parameter for skip responses to the CNI model (hence, the label CNIS model), this modification provides an option to estimate separate *N* parameters for proscriptive and prescriptive norms (i.e., *N*_pro_ and *N*_pre_). It is also possible to estimate separate *C* parameters for the four types of moral dilemmas. By providing an option to estimate separate *N* and *C* parameters for different types of dilemmas, invariance violations can be directly tested within the CNIS model, for example, by testing whether the *N* parameter for proscriptive norms is larger than the *N* parameter for prescriptive norms. To the extent that this is the case, invariance violations can be addressed via the estimation of separate *N* parameters for the two kinds of dilemmas (instead of estimating a single *N* parameter, as it is done in the CNI model).

The option to estimate separate parameters for different kinds of dilemmas is a major advantage of the CNIS model over the original CNI model. Nevertheless, the CNIS model also has some disadvantages. A major limitation is that the CNIS model requires the inclusion of a skip option, which makes it impossible to use the CNIS model for re-analyses of existing data from studies that did not include a skip option. More seriously, the inclusion of a skip option may influence responses in a manner that systematically distorts the outcomes of the task. A potential example might be the paradoxical finding that prescriptive norms have a greater impact than proscriptive norms when participants are allowed to skip dilemmas (Skovgaard-Olsen & Klauer, 2023). Because this finding conflicts with a large body of evidence suggesting the opposite (see Janoff-Bulman et al., 2009), it seems possible that the paradoxical effect is caused by the somewhat unusual inclusion of a skip option. A final concern is that participants are more likely to skip responses on the two types of dilemmas where outcome maximization and norm adherence suggest conflicting choices (see Figure 1). Consistent with this concern, participants in the studies by Skovgaard-Olsen and Klauer (2023) showed the highest proportions of skip responses on dilemmas where (a) a proscriptive norm prohibits action and the benefits of action are greater than the costs and (b) a prescriptive norm prescribes action and the benefits of action are smaller than the costs. Of the total number of trials on which participants chose the skip option in the studies by Skovgaard-Olsen and Klauer (2023), 74.0% came from dilemmas where outcome maximization and norm adherence suggest conflicting choices.^13^ Allowing participants to skip these dilemmas likely undermines reliable measurement of sensitivity to consequences and sensitivity to moral norms with the CNIS model.

### CAN Algorithm

The CAN algorithm was developed to address concerns about the hierarchical structure of the parameters within the CNI model (Liu & Liao, 2021). As noted earlier in this article, the relative positions of the three parameters in the CNI model are arbitrary in the sense that any combinatorially possible arrangement of the three parameters will show the same goodness of fit for a given data set (see Gawronski et al., 2017, 2020). Nevertheless, the numeric scores for the three parameters can differ across model specifications, raising the question of whether the results obtained with the standard model replicate with other model specifications. Although re-analyses of existing data suggest that switching the positions of *C* and *N* does not affect their relations to external variables (see Gawronski et al., 2020), any re-analyses with alternative model specifications involve untestable assumptions about the relative dominance of the three factors. Two additional problems related to the hierarchical structure of the CNI model are that (a) the CNI model is unable to produce a converging solution when participants show a response pattern that either fully conforms to the response pattern captured by the *C* parameter or fully conforms to the response pattern captured by the *N* parameter, and (b) estimates for the *I* parameter are unreliable when participants show a response pattern that can be fully described by a combination of *C* and *N*.

The CAN algorithm resolves these problems by concurrently calculating scores for the three factors in a non-hierarchical manner. Sensitivity to consequences (labeled *C*) is calculated as the difference between the probability of action (vs. inaction) responses on dilemmas where the benefits of action are greater than the costs and the probability of action (vs. inaction) responses on dilemmas where the benefits of action are smaller than the costs. Sensitivity to moral norms (labeled *N*) is calculated as the difference between the probability of action (vs. inaction) responses on dilemmas where a prescriptive norm prescribes action and the probability of action (vs. inaction) responses on dilemmas where a proscriptive norm prohibits action. Finally, general action preferences (labeled *A*) are calculated as the probability of action (vs. inaction) responses across all four types of dilemmas. Note that, while the scoring of the CAN algorithm’s *C* and *N* parameters are directionally consistent with the *C* and *N* parameters of the CNI model, the scoring of the CAN algorithm’s *A* parameter is opposite to the CNI model’s *I* parameter, in that higher scores on the *A* parameter indicate a stronger general preference for action versus inaction.

Because the CAN algorithm provides a tool to concurrently calculate scores for the three factors in a manner that does not include any assumptions about their hierarchical relation, the CAN algorithm has a major advantage over the CNI model. Nevertheless, the CAN algorithm also has two major limitations. First, while the hierarchical structure of the CNI model leads to low correlations between the three parameters, their concurrent calculation in the CAN algorithm often involves high correlations between parameter scores (Ng et al., 2023; Ng, Luke, & Gawronski, 2024). These correlations can lead to either false-positive or false-negative results when the shared variance between parameters is not statistically controlled. A false positive can occur when a significant effect on one parameter produces a spurious effect on another parameter that is due to the shared variance between the two (e.g., Ng et al., 2023). A false negative can occur when a significant effect on one parameter creates systematic error variance on another parameter with shared variance, which can undermine the detection of a qualitatively distinct effect (e.g., Ng, Luke, & Gawronski, 2024). Hence, when testing effects on either of the three CAN parameters, it is critical to always control for the respective other two parameters. Although analyses with the CAN algorithm that did not control for shared variances have occasionally produced results that are different from the ones obtained with the CNI model (e.g., Liu & Liao, 2023), the two approaches almost always produce equivalent results (Liu & Liao, 2021) and discrepancies typically disappear when the shared variance between the three CAN parameters is statistically controlled (e.g., Ng et al., 2023; Ng, Luke, & Gawronski, 2024).

A second limitation of the CAN algorithm that is shared with the CNI model is that the CAN algorithm’s difference scores for *C* and *N* presuppose invariance across dilemma types. Although it remains unclear whether the invariance violations in the modified paradigm with a skip option of Skovgaard-Olsen and Klauer (2023) generalize to the original paradigm without a skip option, the large body of evidence for asymmetries between proscriptive and prescriptive norms (Janoff-Bulman et al., 2009) suggests that concerns about potential invariance violations might be justified. These concerns apply to both the CNI model and the CAN algorithm.

## Open Questions and New Directions

Although the original article on the CNI model was published only 7 years ago (Gawronski et al., 2017), the model has already generated a large amount of research on the determinants and correlates of moral-dilemma responses. Nevertheless, there are still some pertinent questions that need to be addressed. The reviewed work also raises interesting new questions for future research.

### Underlying Mechanisms

The DPM suggests that utilitarian judgments are the product of controlled cognitive analyses of costs and benefits, whereas deontological judgments are rooted in automatic emotional reactions to the idea of causing harm (Greene, 2008). As we explained earlier in this article, research using the CNI model poses a challenge to the DPM. At a minimum, the available evidence suggests that the DPM is incomplete. At worst, it shows that the DPM is incorrect. These issues raise the question of what underlies responses in moral dilemmas. The CNI model does not fill this explanatory gap because it is a descriptive theory that specifies patterns of responses at the behavioral level; it is not a mechanistic theory about mental processes underlying behavioral responses (see Gawronski et al., 2018). As such, the CNI model does not provide answers to the questions of what mental processes underlie sensitivity to consequences, sensitivity to moral norms, and general preference for inaction versus action. Answering this question will be an important task for future research.

A first step in this regard is to account for the available evidence regarding each of the three parameters (see Table 3). What mental process(es) account for the obtained effects on sensitivity to consequences? What mental process(es) account for the obtained effects on sensitivity to moral norms? And what mental process(es) account for the obtained effects on general preference for inaction versus action? Finding answers to these questions seems especially important for the *N* parameter, which is the one that is not accounted for by the DPM, yet is most central for understanding the impacts of various factors (see Table 3) as well as responses in the trolleyology paradigm (see Figure 2). Although our own work has not yet produced a fully developed theory of the processes underlying moral-dilemma judgments, we have some tentative ideas that might serve as a skeleton for future theoretical endeavors.

Regarding the informational underpinnings of sensitivity to consequences, we deem it plausible that people engage in some kind of reasoning process that weighs the costs and benefits of the available options (i.e., action vs. inaction). Yet, a compelling account of sensitivity to consequences likely requires more nuance than the DPM. According to theories invoking the notion of expected utility (e.g., D. J. Cohen & Ahn, 2016), two important components of cost-benefit analyses are (a) the subjective value of the outcomes of a given option and (b) the subjective likelihood that choosing a given option leads to a particular outcome. Arguably, a central aspect of the value component is the number of people whose well-being is affected by a given choice, which is central to the impartiality principle of utilitarian ethics (Kahane et al., 2018) as well as the operationalization of sensitivity to consequences in the CNI model (Gawronski et al., 2020). However, while numbers may be all that matters for subjective assessments of value when the involved individuals are unknown strangers, such assessments are likely more complex when the identities of the involved individuals are known (D. J. Cohen & Ahn, 2016; Hester & Gray, 2020). For example, subjective assessments of value will likely differ depending on whether the target of sacrificial harm is a family member or a known war criminal, even when the number of unknown strangers whose lives are saved are the same. From this perspective, any factor that influences the subjective value of outcomes should influence moral-dilemma responses via sensitivity to consequences. The same is true for the likelihood component, in that any factor that influences the perceived likelihood of desired and undesired outcomes should influence moral-dilemma responses via sensitivity to consequences. To the extent that assessments of value and likelihood can be completed relatively quickly with little cognitive effort (see Trémolière & Bonnefon, 2014), the determinants of perceived value and perceived likelihood may in fact be more important than cognitive reflection. These considerations would explain why effects of cognitive reflection on sensitivity to consequences are relatively small overall (see Figure 6) and why, among the variables investigated in prior studies, (see Table 2) uncertainty about outcomes had by far the largest effect on sensitivity to consequences (Ng et al., 2023).

Regarding the informational underpinnings of sensitivity to moral norms, we already discussed the potential role of negative emotional reactions to the idea of violating moral norms (see Nichols & Mallon, 2006). In terms of the operationalization of sensitivity to moral norms in the CNI model, two central components of this account are (a) thoughts about the proximal effects of a focal choice option (i.e., does it harm or help the target?) and (b) anticipated or experienced emotional reactions to the idea of not choosing the focal option. While the first component represents a precondition for the second, thoughts about the proximal effects of a focal choice option may directly shape sensitivity to moral norms even in the absence of anticipated or experienced emotional reactions (e.g., via an appraisal of the focal choice option for its consistency with normative standards). From this perspective, any factor that influences either of the two components should have corresponding effects on sensitivity to moral norms. This alternative account explains three important findings that seem difficult to reconcile with the DPM. First, the proposed account provides a straightforward explanation for the finding that sensitivity to moral norms is stronger for moral judgments than moral decisions (see Figure 4), in that abstract judgments about the acceptability of a focal choice option are more likely to elicit thoughts about normative standards than concrete decisions about how to respond in a given situation. Second, the proposed account explains why happiness reduces sensitivity to moral norms (see Gawronski et al., 2018), in that happiness may dampen negative emotional reactions to the idea of not choosing a focal option. Finally, the proposed account suggests two potential mechanisms by which cognitive reflection can increase sensitivity to moral norms (see Figure 7), in that cognitive reflection may either (a) enhance thoughts about the proximal effects of a focal choice option or (b) enhance rumination about potentially negative emotional reactions to the idea of not choosing the focal option (or both).

Regarding the informational underpinnings of general preference for inaction versus action, we deem it plausible that negative emotional reactions to the idea of causing harm may contribute to a general aversion against action in moral dilemmas (Gawronski et al., 2017). Yet, action aversion in moral dilemmas may also arise from non-emotional reasoning involving the moral rule “first, do no harm” (Baron & Goodwin, 2020). Either factor should produce a general preference for inaction versus action regardless of the proximal and distal effects of a focal action. A potential third factor that may contribute to a general preference for inaction versus action in moral dilemmas is a domain-independent reluctance to act (Gawronski et al., 2020). Different from the first two cases, action aversion arising from a domain-independent reluctance to act would not be limited to moral dilemmas but generalize to non–moral-decision conflicts. Thus, any factor that influences either of the three determinants should influence general preference for inaction versus action in a corresponding manner.

In sum, while a fully developed mental-process account of the reviewed findings is still lacking, the current review provides a valuable starting point for future theories on the mechanisms underlying moral-dilemma judgments. Although such theories may have superficial resemblance to the DPM, more nuanced assumptions will be required to accommodate various findings that are difficult to reconcile with the DPM. Based on the available evidence, we deem it plausible that (a) sensitivity to consequences is rooted in mental assessments of costs and benefits involving expectancy and value; (b) sensitivity to moral norms is rooted in thoughts about the proximal effects of a focal choice option and anticipated or experienced emotional reactions to the idea of not choosing the focal option; and (c) general preference for inaction versus action can arise from emotional responses to the idea of causing harm, non-emotional reasoning involving the moral rule “first, do no harm,” and a domain-independent reluctance to act.

### Social Impressions

Going beyond traditional applications of the trolleyology paradigm to study moral judgments, an interesting new line of research used the paradigm to investigate social impressions. Instead of asking participants to make judgments about the available options in moral dilemmas, several studies have asked participants to report their impressions of other people who showed a preference for either the utilitarian or the deontological option in moral dilemmas. A common finding in these studies is that hypothetical targets who made deontological judgments are perceived as more trustworthy and having stronger moral character than hypothetical targets who made utilitarian judgments (e.g., Bostyn & Roets, 2017; Everett et al., 2016; Rom et al., 2017; Sacco et al., 2017; Turpin et al., 2021; but see Bostyn et al., 2023; K. M. Smith & Apicella, 2022).

A series of four studies using the CNI model suggests that impressions of morality are shaped by a target’s sensitivity to moral norms with little impact of sensitivity to consequences and general preference for inaction versus action (Gawronski, 2022). In these studies, American MTurk workers were instructed to think about a person they consider morally exceptional and then guess how that person would respond in series of moral dilemmas for research using the CNI model. Participants in various control conditions were asked to do the same for the average person, a specific person with average levels of morality, or a person who is highly influential in society. Results indicated an association between perceived morality and sensitivity to moral norms, in that higher levels of perceived morality were associated with greater sensitivity to moral norms in presumed moral choices. For sensitivity to consequences and general preference for inaction versus action, the findings were mixed and attributable to characteristics confounded with perceived morality (e.g., perceived influence). These results suggest that sensitivity to moral norms is fundamental to perceptions of morality, whereas sensitivity to consequences and general action preferences seem to have little impact on moral impressions. In addition, the findings suggest a hitherto unexplored mechanism underlying moral-dilemma responses by which choices are based on mental simulations of decisions by morally exceptional figures (see Gawronski, 2022).

Expanding on these findings and prior evidence for relatively small associations between political ideology and moral-dilemma responses (Luke & Gawronski, 2021a), we have conducted two CNI model studies to investigate mutual moral perceptions of liberals and conservatives in the United States. To provide a context for these studies, recall that liberals tend to show a stronger sensitivity to consequences in moral judgments than conservatives and that the obtained associations between political ideology and sensitivity to consequences were rather small with correlations between *r* = .12 and *r* = .13 (Luke & Gawronski, 2021a). Political ideology showed no significant associations with sensitivity to moral norms and general preference for inaction versus action. In light of these findings, we were interested in how the available evidence for *actual* differences (similarities) between liberals and conservatives relates to *perceived* differences (similarities) between liberals and conservatives. To this end, we used Prolific Academic’s prescreening filters to recruit balanced samples of self-identified liberals and self-identified conservatives from the United States and presented them with a battery of moral dilemmas by Körner et al. (2020) for research using the CNI model. Participants were asked to indicate whether the typical liberal or the typical conservative would find the described actions acceptable or unacceptable. In a first study, half of the participants were asked to make judgments about the typical liberal, while the other half was asked to make judgments about the typical conservative (see Graham et al., 2012). In a second study, all participants made judgments about both the typical liberal and the typical conservative. Participants in the second study were also asked to indicate if they personally find the described actions acceptable or unacceptable.

For sensitivity to consequences, participants showed no reliable differences in their judgments for the typical liberal and the typical conservative, and this was true regardless of participants’ own political orientation. There were also no reliable differences in terms of general preference for inaction versus action. However, liberal and conservative participants showed a large ingroup bias in the presumed sensitivities to moral norms, in that each group presumed a greater sensitivity to moral norms in judgments about a typical member of their political ingroup compared to a typical member of the political outgroup. Specifically, while liberal participants presumed the typical liberal to show greater sensitivity to moral norms in responses to moral dilemmas than the typical conservative, conservative participants showed the opposite pattern. The size of the obtained ingroup bias ranged from medium to large with effect sizes between *d* = .45 and *d* = .83. Moreover, self-judgments provided by participants in the second study replicated earlier findings by Luke and Gawronski (2021a), in that liberal participants showed a stronger sensitivity to consequences than conservative participants did. However, the two groups did not reliably differ in terms of their sensitivity to moral norms or their general preference for inaction versus action. These results suggest that both liberals and conservatives have distorted perceptions of each other, in that (a) they do not perceive existing differences between liberals and conservatives in terms of their sensitivity to consequences and (b) they perceive differences between liberals and conservatives in terms of their sensitivity to moral norms although there are no such differences. Together with prior findings suggesting a link between perceived morality and sensitivity to moral norms (Gawronski, 2022), the findings suggest that both liberals and conservatives perceive their political ingroup as morally superior to the political outgroup. Considering the increasing levels of affective polarization in the United States (Finkel et al., 2020), these findings raise important questions about the factors that shape perceived moral superiority and its potential consequences on actions against political outgroup members. An ironic aspect of perceived moral superiority of one’s political ingroup is that it may serve to justify immoral actions against outgroup members, including online harassment and physical assault. Future research using the CNI model to quantify perceived moral superiority of one’s political ingroup may help to provide a deeper understanding of how moral perceptions may contribute or exacerbate political conflict and affective polarization.

### Applications

A small number of trolleyology studies have linked differences in moral-dilemma responses to clinically relevant traits, including autism spectrum disorder and alexithymia (e.g., Gleichgerrcht et al., 2013; Patil et al., 2016). Across these studies, participants with a clinical diagnosis of autism or alexithymia showed a stronger preference for utilitarian over deontological judgments than neurotypical participants. These associations correspond to the one obtained for psychopathic traits, in that higher levels of psychopathy have been found to be associated with a stronger preference for utilitarian over deontological judgments in the trolleyology paradigm (for a meta-analysis, see Marshall et al., 2018). Although the defining characteristics of autism, alexithymia, and psychopathy are rather different, extant accounts attribute the observed associations to the same underlying construct, suggesting that deficits in empathy lead to a stronger preference for utilitarian over deontological judgments among individuals with elevated levels of autism, alexithymia, or psychopathy (cf. Patil et al., 2016; Patil & Silani, 2014; Takamatsu & Takai, 2019).

By distinguishing between multiple distinct factors underlying moral-dilemma responses, the CNI model permits a more nuanced analysis of commonalities and differences between distinct clinically relevant traits. Although it is possible that individuals with elevated levels of autism, alexithymia, and psychopathy share certain characteristics in their psychological profiles, it seems rather unlikely that associations of the three traits with moral judgments have the same mental underpinnings. Thus, prior studies using the CNI model to investigate associations between moral-dilemma responses and psychopathy (e.g., Gawronski et al., 2017; Körner et al., 2020; S. Li et al., 2020; Luke et al., 2022; Luke & Gawronski, 2021b; Ng et al., 2022; Paruzel-Czachura & Farny, 2024) could serve as a valuable starting point for future research on associations with autism and alexithymia. Such studies may not only provide a deeper understanding autism and alexithymia; they may also contribute to a better understanding of the mental processes underlying sensitivity to consequences, sensitivity to moral norms, and general preference for inaction versus action.

The obtained associations between psychopathy and different factors underlying moral-dilemma responses may also serve as a basis for potential applications in forensic contexts. In an ongoing collaborative project, we are exploring potential differences in moral-dilemma responses between incarcerated violent offenders, incarcerated non-violent offenders, and non-incarcerated individuals. To the extent that the three groups show unique profiles in their sensitivity to consequences, sensitivity to moral norms, and general preference for inaction versus action, an interesting follow-up question is whether response profiles identified with the CNI model could be used to predict recidivism and how prediction accuracy of the CNI model compares to extant prediction tools in forensic diagnostics (e.g., Hart & Hare, 1989). Although much more research is needed before the CNI model can be used as a diagnostic instrument in forensic contexts, a major advantage of the CNI model to traditional approaches is that there is much more ambiguity about what would constitute a “desirable” or “correct” response in the CNI model dilemmas, which should make them less susceptible to socially desirable responding. Studies using the CNI model with forensic samples may serve as a valuable starting point for potential diagnostic applications.

### Advanced Algorithm

A final issue concerns known limitations of the CNI model. Although we discussed several reasons why including a skip option for applications of the CNIS model (Skovgaard-Olsen & Klauer, 2023) may be problematic, the concern about potential invariance violations arising from asymmetric effects of proscriptive and prescriptive norms (see Janoff-Bulman et al., 2009) seems justified and should be taken seriously. Another important concern pertains to the arbitrary hierarchical structure of the parameters within the CNI model. Although the latter issue can be addressed by cross-validating findings with the CAN algorithm (Liu & Liao, 2021), the CAN algorithm also has some known limitations. One limitation is the high intercorrelation between the CAN parameters, which can lead both false positives and false negatives when the shared variances between parameter are not statistically controlled (Ng et al., 2023; Ng, Luke, & Gawronski, 2024). Another limitation that is shared with the CNI model is that the CAN algorithm’s difference scores for *C* and *N* presuppose invariance across dilemma types. A more efficient way to address the known limitations of the CNI model would be an alternative statistical approach that captures the three factors without assuming invariance and a particular hierarchical structure. We are currently working on a statistical solution to this problem.

## Constraints on Generality

Regarding the generality of the reviewed findings, it is worth distinguishing between two levels of analysis. The first level involves the application of the CNI model to analyze moral-dilemma responses. The second level involves investigations of effects on and associations with the three factors captured by the CNI model. Generality claims at the first level of analysis are supported by the large number of studies that successfully used the CNI model with samples from four continents (i.e., Asia, Europe, North America, Oceania) and various countries including Australia (e.g., Halliwell, 2021), China (e.g., S. Li et al., 2020), Germany (e.g., Kroneisen & Heck, 2020), Iran (e.g., Barabadi et al., 2023), Japan (e.g., Qian et al., 2023), Poland (e.g., Paruzel-Czachura et al., 2023), the United Kingdom (e.g., Luke & Gawronski, 2021a), and the United States (e.g., Gawronski et al., 2017). The CNI model has also been successfully used with university students (e.g., Zhang et al., 2018), participants recruited from the local community (e.g., Paruzel-Czachura et al., 2023), and workers on various crowdsourcing platforms such as Amazon’s MTurk (e.g., Gawronski et al., 2017), Prolific Academic (e.g., Ng et al., 2022), CloudResearch (e.g., Luke & Gawronski, 2021a), Lancers (Speckmann et al., 2024), SurveySwap (e.g., Oldham, 2022), and Questant (e.g., Qian et al., 2023). Although groups with distinct social identities may differ in terms their sensitivity to consequences, sensitivity to moral norms, and general preference for inaction versus action (e.g., Gawronski et al., 2017; Qian et al., 2023; Speckmann et al., 2024), such differences do not question the validity of the CNI model as a computational approach to quantify the three factors underlying responses to moral dilemmas.

Generality claims at the second level of analysis vary substantially in terms of their supportive evidence. While some of the reviewed effects have been replicated with samples from multiple countries and cultures, others have been demonstrated in only a single study with highly homogeneous samples. For example, associations of the three CNI model parameters with psychopathy have been studied in a variety of samples from the United States (Luke et al., 2022), Poland (Paruzel-Czachura & Farny, 2024), and China (S. Li et al., 2020), and with participants recruited from the local community (e.g., Paruzel-Czachura & Farny, 2024) and crowdsourcing platforms such as Amazon’s MTurk (e.g., Gawronski et al., 2017) and Prolific Academic (e.g., Ng et al., 2022). In contrast, associations of the three CNI model parameters with testosterone levels have been tested in only one study with undergraduate students from the United States (Brannon et al., 2019). Claims about the generality of effects on and associations with the three factors captured by the CNI model should be evaluated based on the diversity of the populations in which they have been demonstrated.

A final point pertains to the demarcation between moral and non-moral issues and its implication for the generality of the CNI model. All dilemmas for research using the CNI model involve decisions about care and harm (Gawronski et al., 2017; Körner et al., 2020); the available set of CNI dilemmas does not include any scenarios about other moral issues (e.g., justice and fairness). While some have argued that harm is fundamental to morality and that there are no harmless wrongs (Schein & Gray, 2018), others claimed that morality is not unitary but instead involves multiple psychologically distinct moral foundations, including care, fairness, loyalty, authority, and purity (Graham et al., 2013). From a purely technical view, it might be possible to create sets of moral dilemmas for other dimensions of morality and analyze responses to those dilemmas with the CNI model. However, a practical obstacle to any such endeavor is that consensus about moral norms and consequences for the greater good is much smaller in domains that do not involve care and harm (Graham et al., 2009). Lack of consensus in these domains makes it inherently difficult to develop construct-valid manipulations of moral norms and consequences for greater good, which is essential for applications of the CNI model. Thus, although the CNI model may be well-suited to study responses in moral dilemmas involving issues of care and harm, it is less suited to study moral judgments in other domains.

## Conclusions

Trolleyology research has been criticized for several reasons, including the implausible and unrealistic nature of the employed dilemmas (e.g., Bauman et al., 2014; Körner et al., 2019), the confounded measurement of utilitarian and deontological tendencies (Conway & Gawronski, 2013), and the conflation of moral codes with general action tendencies (Crone & Laham, 2017). The CNI model addresses these issues by disentangling sensitivity to consequences, sensitivity to moral norms, and general preference for inaction versus action in responses to plausible dilemmas based on real-world events (Gawronski et al., 2017; Körner et al., 2020). Although the CNI model has known limitations that should be addressed, research using the model has provided valuable insights into the determinants and correlates of responses in moral dilemmas. Moreover, while the CNI model itself is not in conflict with prominent theories about the processes underlying moral-dilemma responses, various findings obtained with the CNI model are difficult to reconcile with these theories. Thus, a major challenge for future work is to develop an alternative mechanistic account that can explain the reviewed findings and ideally generate novel predictions about determinants and correlates of the three factors underlying moral-dilemma responses. While this challenge remains to be addressed, the available evidence provides a basis for using the CNI model in applied areas concerned with moral judgments, including clinical and forensic contexts. By disentangling the roles of multiple factors in responses to moral dilemmas, the CNI model offers a more nuanced understanding of moral-dilemma responses than the controversial trolleyology paradigm.

## Appendix

### CNI Model Equations

Model equations for the estimation of sensitivity to consequences (*C*), sensitivity to moral norms (*N*), and general preference for inaction versus action (*I*) in responses to moral dilemmas with proscriptive versus prescriptive norms and benefits of action for overall wellbeing that are either greater or smaller than the costs of action for wellbeing. Reproduced from the study by Gawronski et al. (2017). Reprinted with permission from the American Psychological Association.

*p*(inaction| proscriptive norm, benefits > costs) = ([1 − *C*] × *N*) + ( [1 − *C*] × [1 − *N*] × *I*)

*p*(inaction| proscriptive norm, benefits < costs) = *C* + ([1 − *C*] × *N*) + ( [1 − *C*] × [1 − *N*] × *I*)

*p*(inaction| prescriptive norm, benefits > costs) = (1 − *C*) × (1 − *N*) × *I*

*p*(inaction| prescriptive norm, benefits < costs) = *C* + ([1 − *C*] × [1 − *N*] × *I*)

*p*(action| proscriptive norm, benefits > costs) = *C* + ([1 − *C*] × [1 − *N*] × [1 − *I*])

*p*(action| proscriptive norm, benefits < costs) = (1 − *C*) × (1 − *N*) × (1 − *I*)

*p*(action| prescriptive norm, benefits > costs) = *C* + ([1 − *C*] × *N*) + ([1 − *C*] × [1 − *N*] × [1 − *I*])

*p*(action| prescriptive norm, benefits < costs) = ([1 − *C*] × *N*) + ([1 − *C*] × [1 − *N*] × [1 − *I*])

## Notes

1. Because *p*(action) = 1 − *p*(inaction), the four equations for inaction responses presented in the Appendix are redundant with the four equations for action responses.

2. A tutorial on how to conduct group-level comparisons with the CNI model has been provided by Gawronski et al. (2017) at https://osf.io/xt66w/; a tutorial on how to conduct individual-difference analyses with the CNI model has been provided by Körner et al. (2020) at https://osf.io/ndf4w/.

3. The data and analysis code for the reported analyses are available at https://osf.io/2pdnz/.

4. The differences between the corresponding zero-order correlations are statistically significant for both the *C* parameter (*Z* = 2.43, *p* = .015) and the *N* parameter (*Z* = 3.21, *p* = .001).

5. The difference between the corresponding zero-order correlations with the *I* parameter is statistically significant (*Z* = 2.00, *p* = .045).

6. Regarding the small size of the obtained associations, it is worth noting that the switch and footbridge dilemmas each include only a single observation, which makes them highly susceptible to measurement error (see Dang et al., 2020). Associations with the three CNI parameters tend be much larger in analyses using aggregate data from multiple dilemmas that are structurally equivalent to the trolley problem (see Gawronski et al., 2020).

7. Two of the citations in Google Scholar came from pseudo-articles whose links led to scam websites.

8. We review these works in later sections of this article when we discuss alternative approaches and new directions for future research.

9. The study by Paruzel-Czachura and Farny (2024) included three distinct measures of psychopathy: the Levenson Self-Report Psychopathy Scale (LSRP; Levenson et al., 1995), the Psychopathic Personality Inventory-Revised (PPI; Lilienfeld & Widows, 2005), and the Triarchic Psychopathy Measure (TriPM; Patrick, 2010). Because including multiple effect sizes from the same sample would violate the independent effect-size assumption (see Lipsey & Wilson, 2001), we used a random procedure to select one of the three psychopathy scales for inclusion in the current meta-analysis.

10. The demographic questionnaire in the study by Gawronski et al. (2017) included only the two binary response options: female and male.

11. Stankovich et al. (2022) report a meta-analytic odds ratio of 1.64 for personal dilemmas and a meta-analytic odds ratio of 1.08 for impersonal dilemmas. According to calculations by Chen et al. (2010), odds ratios of 1.68, 3.47, and 6.71 correspond to Cohen’s *d*s of 0.2 (small effect), 0.5 (medium effect), and 0.8 (large effect), respectively.

12. Baron and Goodwin (2020) used the process dissociation model of Conway and Gawronski (2013), not the CNI model of Gawronski et al. (2017). We explain the difference between the two models in the section on alternative approaches.

13. A re-analysis of the data from Skovgaard-Olsen and Klauer (2023) revealed that of the trials on which participants skipped responses, 37.2% came from dilemmas with a proscriptive norm where the benefits of action were greater than the costs, 12.3% came from dilemmas with a proscriptive norm where the benefits of action were smaller than the costs, 13.7% came from dilemmas with a prescriptive norm where the benefits of action were greater than the costs, and 36.8% came from dilemmas with a prescriptive norm where the benefits of action were smaller than the costs.
